# Shifts in microbial communities in soil, rhizosphere and roots of two major crop systems under elevated CO_2_ and O_3_

**DOI:** 10.1038/s41598-017-14936-2

**Published:** 2017-11-03

**Authors:** Peng Wang, Ellen L. Marsh, Elizabeth A. Ainsworth, Andrew D. B. Leakey, Amy M. Sheflin, Daniel P. Schachtman

**Affiliations:** 1 Department of Agronomy and Horticulture, University of Nebraska Lincoln, Lincoln, NE 68588 USA; 2 Department of Plant Biology, University of Illinois at Urbana-Champaign, Urbana, IL 61801 USA; 3 USDA ARS Global Change and Photosynthesis Research Unit, Urbana, IL 61801 USA; 4 Proteomics and Metabolomics Facility, Colorado State University, Fort Collins, CO 80523 USA

## Abstract

Rising atmospheric concentrations of CO_2_ and O_3_ are key features of global environmental change. To investigate changes in the belowground bacterial community composition in response to elevated CO_2_ and O_3_ (eCO_2_ and eO_3_) the endosphere, rhizosphere and soil were sampled from soybeans under eCO_2_ and maize under eO_3_. The maize rhizosphere and endosphere α-diversity was higher than soybean, which may be due to a high relative abundance of Rhizobiales. Only the rhizosphere microbiome composition of the soybeans changed in response to eCO_2_, associated with an increased abundance of nitrogen fixing microbes. In maize, the microbiome composition was altered by the genotype and linked to differences in root exudate profiles. The eO_3_ treatment did not change the microbial communities in the rhizosphere, but altered the soil communities where hybrid maize was grown. In contrast to previous studies that focused exclusively on the soil, this study provides new insights into the effects of plant root exudates on the composition of the belowground microbiome in response to changing atmospheric conditions. Our results demonstrate that plant species and plant genotype were key factors driving the changes in the belowground bacterial community composition in agroecosystems that experience rising levels of atmospheric CO_2_ and O_3_.

## Introduction

Research has shown that changes in the atmospheric concentrations of carbon dioxide (CO_2_) and ozone (O_3_) alter crop growth and yields^1,2^. From 1960 to 2015, atmospheric CO_2_ concentrations have increased from 320 ppm to 400 ppm and are expected to reach 700 ppm by 2100 (www.co2.earth). Ground-level O_3_ concentrations are increasing between 1–2% per year as a byproduct of anthropogenic nitrous oxide and may be as high as 70 ppb by 2100^3^. Photosynthetic CO_2_ assimilation, biomass production and soybean yield (*Glycine max* L.) are stimulated by elevated CO_2_ when water supply is adequate^4,5^. In contrast, elevated O_3_ causes oxidative stress that decreases photosynthetic CO_2_ assimilation, biomass production and crop yields (e.g., maize, *Zea mays* ^2,6^) and indirectly influences the belowground microbial communities.

Many features of short-and long-term plant-microbe-soil interactions are modified by eCO_2_ and/or eO_3_, resulting in altered plant carbon exudation or deposition as well as changes in plant nutrient status and water relations^7–11^. There is evidence across diverse ecosystems for elevated CO_2_ and/or O_3_ effects on: (1) the quantity and quality of roots^12,13^, root exudates^14^ and leaf litter^15^ as inputs to the soil; (2) plant mineral nutrient uptake^16,17^, nitrogen fixation by symbiotic bacteria^18^ and soil weathering, organic matter decomposition and nutrient acquisition by mycorrhizae^19–21^ and (3) the extent and location of water use in the soil profile^5^. Consequently, these treatments can modify soil N mineralization, organic content, C storage, respiration^22,23^, N_2_O emissions^24^, and even the physical structure^25^ with consequences for ecosystem-scale biogeochemistry^11,26–28^. Despite the recognized importance and sensitivity of the soil and plant microbiome to environmental perturbations^29,30^, little is known about the microbe community response and the role of soil microbes in mediating plant and ecosystem responses to eCO_2_ and eO_3_.

Many factors determine the microbial response to elevated greenhouse gases. Elevated CO_2_ is known to alter the quality and quantity of litter and soil carbon, which are dependent on the nutrient status of the system. In a long-term study in low-nutrient grasslands, changes in soil microbial communities due to eCO_2_ may have been due to reductions in total nitrogen and carbon^31^. In another long-term study on nitrogen rich soils involving a wheat-soybean rotation, CO_2_, but not O_3_, had a significant effect on microbial communities^32^. Soil microbial biomass was enhanced and composition altered in that study, but could only be detected in years 3 and 4. In contrast to the grasslands study changes in composition were ascribed to enhanced N and C availability. Other studies showed that the effect of eCO_2_ on N and C properties in soil is highly dependent on the plant species^33^. To summarize, previous studies have found that changes in microbial communities are sensitive to time, soil nutrient status and plant community composition.

Microbial communities in soils are also structured by the genotypic variation in plant carbon and nutrient content and water status. A small but significant proportion of the total bacterial diversity in the maize and rice rhizospheres has been ascribed to plant genotypic variation, a potentially heritable trait^34,35^. In *Arabidopsis*, the ecotype or genotype also controls certain root-inhabiting microbes at different developmental stages and in mutants with different phytohormones levels^36–38^. Although studies have shown that plant genotype and/or species determine a significant amount of the soil, rhizosphere and endosphere microbiome variation, the mechanisms underlying this variation are poorly understood^37,39–42^.

To determine the effects of atmospheric change on the microbial community composition and diversity in the soil, rhizosphere and endosphere, soybean and maize were grown in eCO_2_ and eO_3_ conditions, respectively, at the Free Air Concentration Enrichment (FACE) facility located in Champaign, Illinois. Both of these crops were also grown under ambient conditions to serve as a control for the experiment. Recent studies using this system showed that the functional structure and metabolic potential of soil microbe communities can be altered^43,44^ such that carbon and nitrogen cycling were stimulated under eCO_2_, while_,_ N cycle processes were inhibited under eO_3_ in soybean fields. However, these previous studies did not investigate the changes in the endosphere and rhizosphere, where plant-microbe interactions are strongest and might be expected to show the strongest response to treatment. In our study, DNA was extracted from root, rhizosphere and soil samples and bacterial sequences were amplified using V4 primers for the 16S gene ^45^ followed by next generation sequencing of pooled amplicons. Distinct differences in the bacterial community composition, attributed to the treatment regime and host species genotype, were observed. This study provides new insights into the response of the soil microbiome to eCO_2_ and eO_3_ in the dominant C_3_ and C_4_ agroecosystems in the U.S.

## Results

### Diversity in maize and soybean

To directly compare the α-diversity between maize and soybean samples with differing sequence depths, the data were rarefied to the same depth using QIIME (Fig. S1). Measures of α-diversity, using the Shannon Diversity Index, revealed a decreasing diversity gradient from the soil to the root in both the maize and soybean samples (Table 1a,b,c). Endosphere, rhizosphere (RHZ) and soil microbial communities showed the lowest, intermediate, and highest α-diversity values, respectively (Table 1). The diversity of microbial communities differed significantly by crop species, with the maize endosphere and rhizosphere showing a greater diversity of microbial OTUs (operational taxonomic units), compared with soybean (Table 1a). eCO_2_ significantly decreased the species diversity in soybean RHZ. Under eO_3_ conditions, no change in α-diversity was detected in maize endosphere, RHZ or soil (Table 1b). However, a genotype comparison showed differences in the soil microbial diversity under aO _3_ (ambient ozone) and in the RHZ under eO_3_ (Table 1c).

**Table 1:** Changes in α-diversity in the endosphere, rhizosphere and soil of maize and soybean.

Sample type	a		b				c			
	Soy	Maize	Soy		Maize		Maize			
			aCO_2_	eCO_2_	aO_3_	eO_3_	aO_3_		eO_3_	
							B73	B73xMo17	B73	B73xMo17
Endosphere	2.55e	5.23d	3.01	2.13	5.22	5.23	5.32	5.12	5.15	5.32
Rhizosphere	8.26c	9.03b	8.58**	7.93**	8.96	9.11	8.87	9.05	8.92*	9.29*
Soil	9.71a	10.03a	9.91	9.50	10.02	10.05	10.15**	9.88**	10.09	10.01

(a) Shannon index for mean α-diversity of the microbial communities of endosphere, RHZ and soil in both maize and soybean. Different letters indicate significant differences (p ≤ 0.05) as determined by nonparameter Wilcoxon test. (b) Shannon index for α-diversity of the microbial communities according to treatments ambient/elevated a/eO_3_ and a/eCO_2_ on different sample types in maize and soybean. Significant differences between treatment within each sample type by one-way ANOVA are indicated by ‘**’*P* ≤ 0.01, ‘*’ *P* ≤ 0.05. (c) Shannon index compared between maize genotypes. Significant differences between genotypes within each sample type by nonparameter Wilcoxon test are indicated by ‘**’ *P* ≤ 0.01, ‘*’ *P* ≤ 0.05.

### β-Diversity in maize and soybean

**Figure 1:**
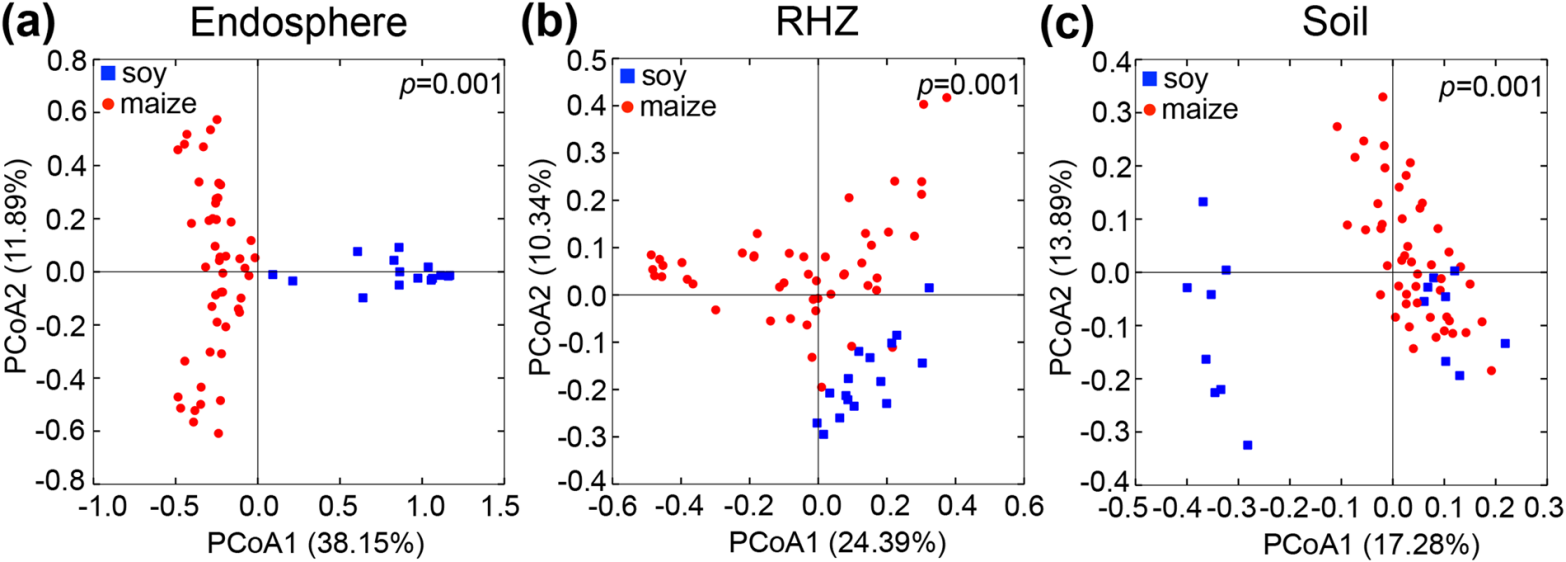
Microbe community composition (β-diversity) differs between maize and soybean in (**a**) endosphere (**b**) RHZ and (**c**) soil microbial communities from ambient atmospheric conditions. Principal coordinate visualization (PCoA) using the weighted UniFrac distance (WUF) matrix shows significant difference between microbial community composition based on crop species grown as detected by permutational MANOVA in each sample type (*p* = 0.001).

Unconstrained principal coordinate analyses (PCoAs) of weighted (WUF) and unweighted (UUF) UniFrac^46^ distance matrices were performed to estimate β-diversity. In the WUF and UUF analyses, the soybean and maize communities of plants grown under ambient conditions separated across the first principal coordinate, indicating that the largest source of variation in the different sample types could be ascribed to the plant species (Fig. 1, WUF and Fig. S2, UUF). The endosphere samples were distinctly different from the soil and RHZ, which appeared to have overlapping microbial communities. Permutational multivariate analysis of variance (PERMANOVA) showed that there was a significant difference between the soybean and maize bacterial communities under ambient conditions in endosphere, RHZ and soil when tested using either WUF or UUF matrices (Fig. 1, WUF and Fig. S2, UUF).

### Elevated CO_2_ alters the microbial communities in soybean

**Figure 2:**
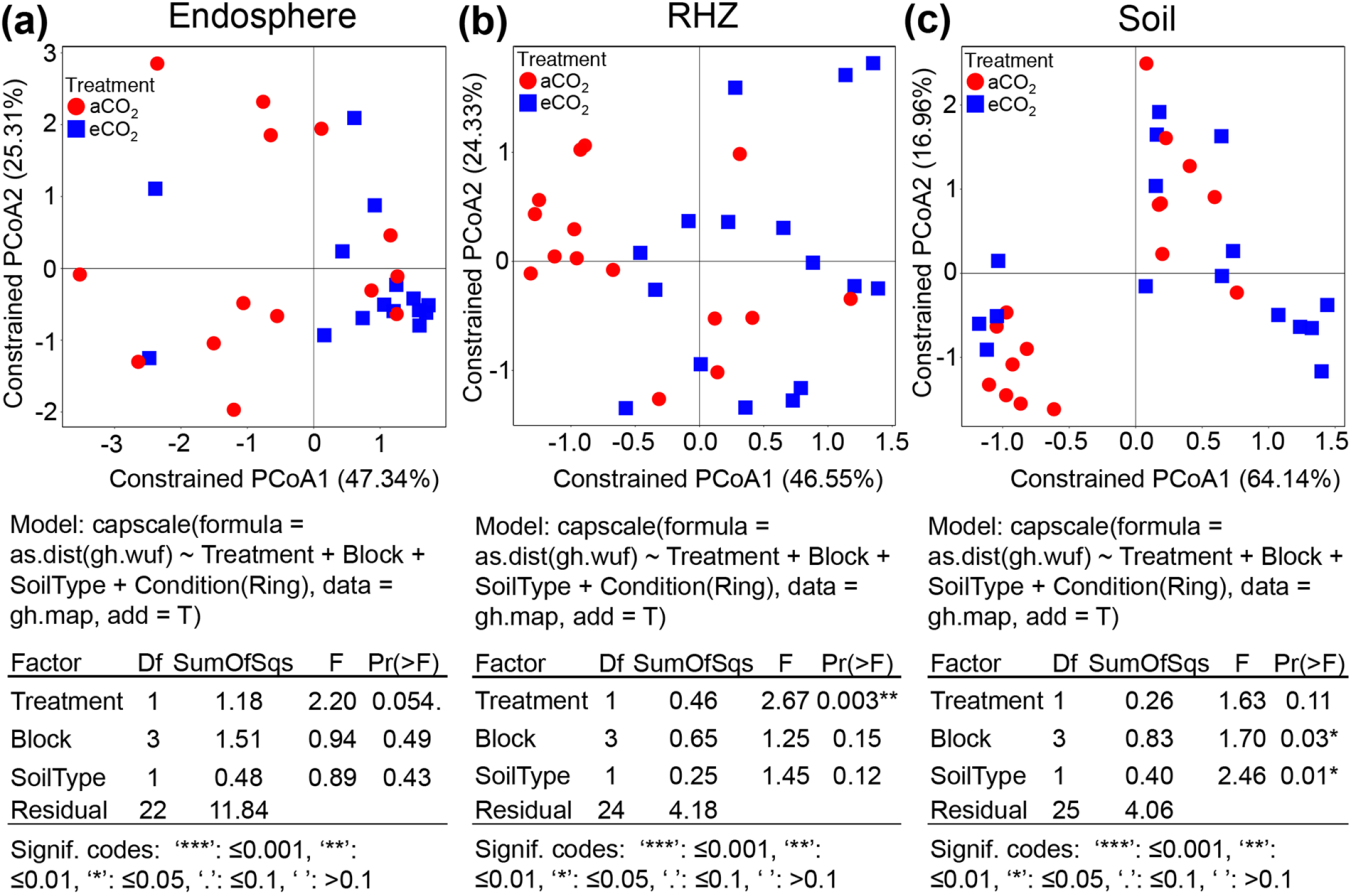
Principal coordinate graph and CAP analysis of soybean using weighted UniFrac matrix of microbial communities from ambient aCO_2_ and elevated eCO_2_ conditions. Permutational MANOVA was generated using a model constrained for treatment, block and soil type. Sample types are separated for CAP analysis of the microbial community structure in (**a**) endosphere (**b**) RHZ and (**c**) soil of soybean using UniFrac weighted matrix.

Canonical analysis of principal coordinates (CAP) was performed to assess how each experimental variable, including soil type and sequencing method, contributed to the variation in β-diversity. The results from this analysis revealed that the microbial community composition was significantly different among sample types using both WUF (p < 0.001, 7.8% variation explained) and UUF (p < 0.001, 3.2% variation explained) distance matrices. Moreover, there was a significant interaction between sample type and treatment (p < 0.009 by WUF and p < 0.001 by UUF) (Fig. S3) and therefore, each sample type (i.e., endosphere, RHZ, and soil) was analyzed individually . When each sample type was analyzed individually, the effect of the treatment on the endosphere samples approached statistical significance (p = 0.054) (Fig. 2a). In the RHZ there were significant differences in microbial community composition in aCO_2_, (ambient CO_2_) compared to eCO_2_ (p = 0.003, WUF, Fig. 2b and p = 0.003, UUF, Fig. S4b).

In the soil samples, significant differences were not detected between treatments, but soil type (i.e., Drummer silty clay loam and Flanagan silt loam) and block had a significant impact on microbial communities (Fig. 2c, WUF, block p = 0.03, 15.0% variation explained: soil type p = 0.01, 7.2% variation explained). Furthermore, the relative abundance of microbes related to the nitrogen cycling were significantly enriched in the Flanagan silt loam (Fig. S5) including microbes from Bradyrhizobium, Mesorhizobium, and Phyllobacteriaceae.

When the UUF matrix was used for analysis, soil type and block were also identified as significant sources of variation in microbial communities in the RHZ samples (block p = 0.03, 12.3% variation explained, soil type p = 0.02, 4.8% variation explained). This result indicated that soil type influenced the microbial community composition in the soil and RHZ samples (Fig. 2b,c, WUF and Figs S4b and S4c), highlighting the need for a replicated block design and soil type characterization. This finding was unexpected as the chemical characteristics of the soils in under different treatments regimes were similar (Dataset S1).

To identify differences in RHZ OTUs between aCO_2_ and eCO_2_ conditions, we conducted a differential OTU abundance analysis using DESeq. 2 in QIIME. Based on an adjusted P value cutoff of 0.05, there were 496 OTUs that were significantly enriched or depleted in the RHZ samples under eCO_2_ conditions (Dataset S2). These OTUs included members of 68 families and were dominated by Comamonadaceae (aCO_2_: 9.9%, eCO2: 10.4%), Sphingomonadaceae (aCO_2_: 6.0%, eCO_2_: 4.8%), Gaiellaceae (aCO_2_: 2.7%, eCO_2_: 2.4%) and Streptomycetaceae (aCO_2_: 4.6%, eCO_2_: 4.1%) (Fig. S6).

### Maize microbial communities vary by sample type, genotype and treatment

**Figure 3:**
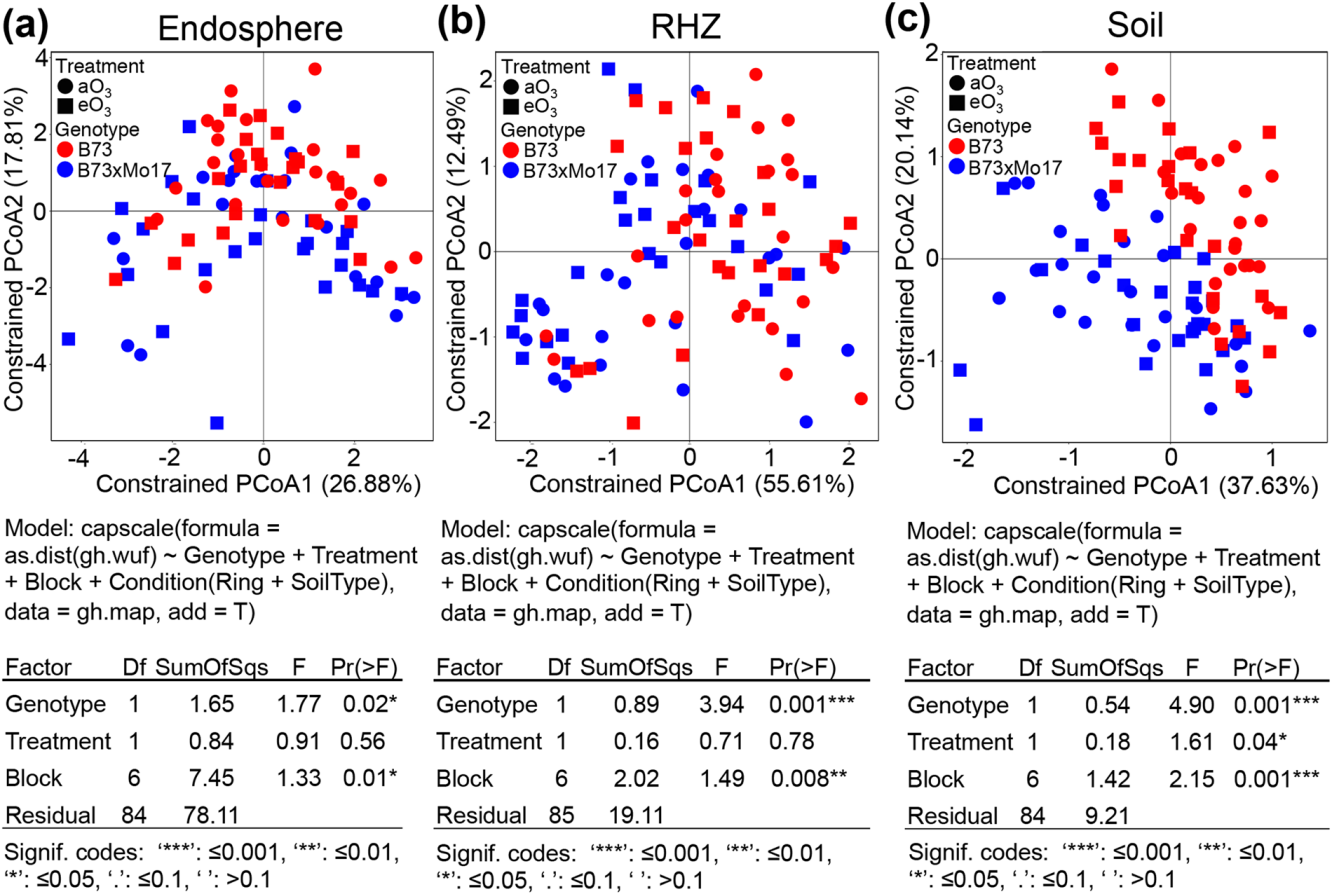
Principal coordinate visualization of maize sample type, genotype and treatment grown under ambient aO_3_ and elevated eO_3_. CAP analysis was completed using the WUF matrix. Sample types are separated for CAP analysis of the microbial community’s composition in (**a**) endosphere (**b**) RHZ and (**c**) soil of maize. Genotype significantly affects the microbial communities in all the sample types, and treatment only significantly affects the microbial communities in bulk soil.

We examined the microbial community composition in two maize genotypes, inbred B73 and hybrid B73 x Mo17, in different sample types (endosphere, RHZ and soil), as well as in the two treatment regimes. CAP was used to discern which factors (i.e., sample type, genotype, and treatment) contributed to the variation in β-diversity. Results revealed that the microbial community structure was significantly different between each sample type and genotype using WUF and UUF matrices (Figs S7a, b, d) and therefore, each sample type was analyzed individually. WUF and UUF analyses revealed that microbial community composition in the endosphere, RHZ and soil were significantly different between maize genotypes in both WUF and UUF analyses (Figs 3a,b,c and S8). Results from UUF analysis also revealed that the microbial community composition in aO_3_ (ambient ozone) and eO_3_ conditions was also significantly different in the endosphere (p = 0.03) and soil (p = 0.01) but not in the RHZ (p = 0.89) (Fig. S8). The microbial community composition was only significantly different in the soil (p = 0.04) in WUF distance analysis (Fig. 3). These results showed that ozone concentration only significantly affected the microbial community composition in the endosphere and soil.

In contrast to our findings in soybean the two soil types in this experiment (Drummer silty clay loam and Flanagan silt loam) didn’t significantly influence the microbial communities (Fig. S9). However, the effect of the block on the microbial community composition was significant in both WUF or UUF analysis (Fig. S9). These results indicate that block and genotype were the two most important variables that altered the microbial community composition where maize was grown in these experiments.

### Inbred and hybrid maize respond differently to eO_3_ – treatment effects on each genotype

Due to the significant differences in β-diversity between ambient O_3_ and elevated O_3_ (Fig. S7f), the effects of eO_3_ on microbial communities in different maize genotypes were further analyzed individually by sample type (Fig. S10). In the endosphere samples, there were no significant differences in the microbial community composition between eO_3_ and aO_3_ treatments in B73 x Mo17. However, significant differences were detected in B73 using the UUF matrix (p = 0.001, Fig. S10j). The microbial communities of the RHZ in B73 x Mo17 and B73 were not significantly different under eO_3_ conditions, consistent with the CAP analysis (Figs 3b, S8b,S9b,e). A significant difference in the soil microbial communities was detected between eO_3_ and aO_3_ conditions in B73 x Mo17 by both UUF and WUF analyses (UUF, p = 0.004; WUF, p = 0.013). However, no significant difference could be detected where the inbred B73 was grown (Fig. S10 c, f, i, l).

### Host influence on microbiome in maize may be mediated by root exudates – genotype effects in each treatment

**Figure 4:**
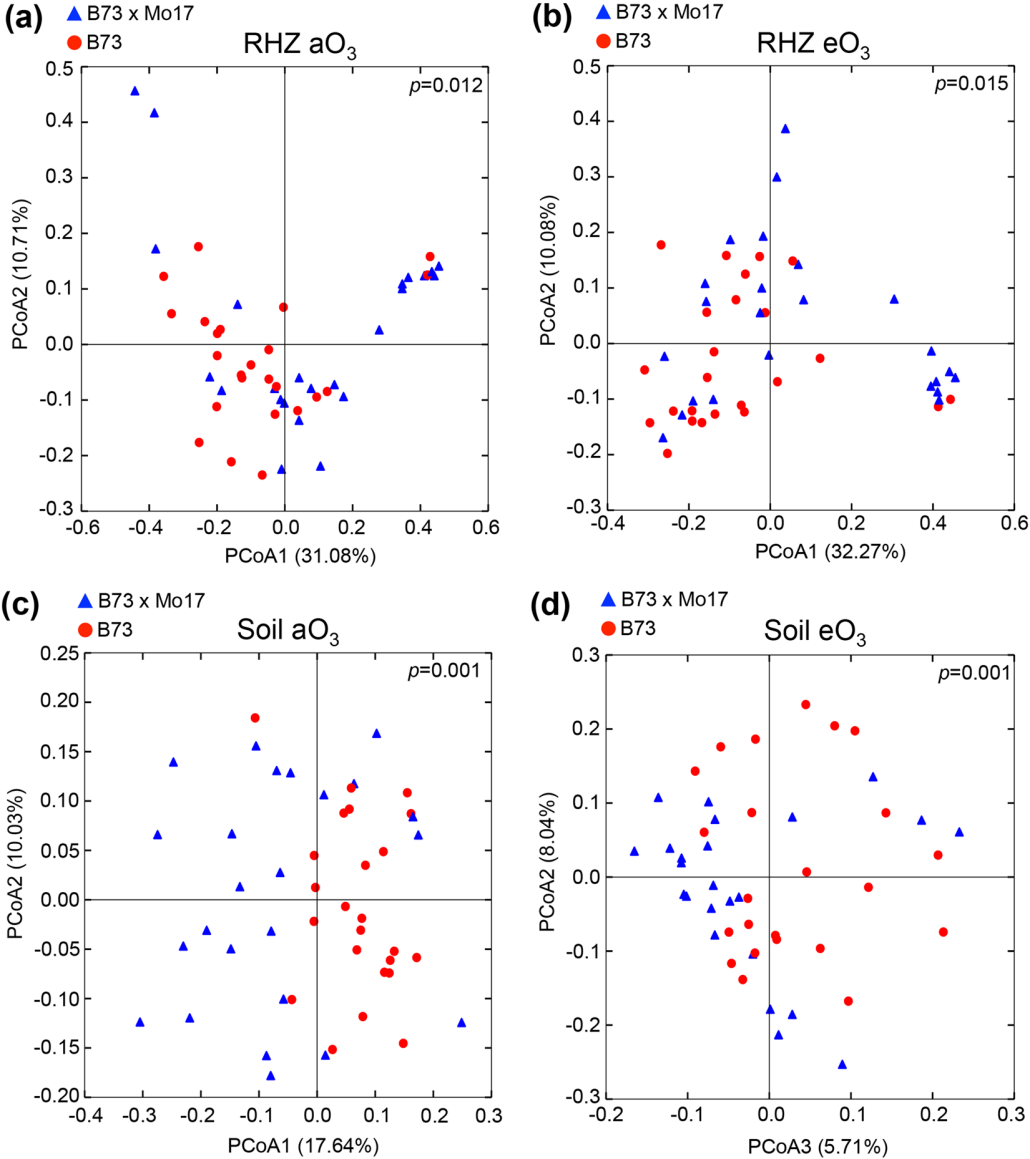
Principal coordinate visualization and analysis (WUF) of genotypic effect on microbial communities for the maize hybrid B73 x Mo17 and inbred B73 in (**a**) RHZ under ambient aO_3_ conditions, (**b**) RHZ under elevated eO_3_ conditions, (**c**) soil under ambient aO_3_ conditions, (**d**) soil under elevated eO_3_ conditions. Blue triangles indicate the B73 x Mo17 and red circles indicate B73.

To determine if the strong observed genotypic influence on microbial community composition was an inherent genetic property or caused by environment conditions, the aO_3_ and eO_3_ data were analyzed separately. In the endosphere, a significant difference between the microbial community composition in B73 and B73 x Mo17 was detected in the aO_3_ (p = 0.012) and eO_3_ datasets (p = 0.001) using the UUF matrix (Fig. S11c, f). The RHZ microbial community compositions of the inbred B73 and hybrid B73 x Mo17 under aO_3_ and eO_3_ conditions were significantly different using the WUF matrix (Fig. 4a,b) but not detected by the UUF analysis (Fig. S11d, g). In aO_3_ and eO_3_ conditions, significant differences were detected in the soil microbial communities of B73 and hybrid B73 x Mo17 (Fig. 4c,d, WUF, aO_3_: p = 0.001, eO_3_: p = 0.001). Further analysis with the UUF matrix detected a significant difference between the soils in which the genotypes were grown (p = 0.002) under aO_3_ conditions but not under eO_3_ (p = 0.059) (Fig. S11e,h).

**Figure 5:**
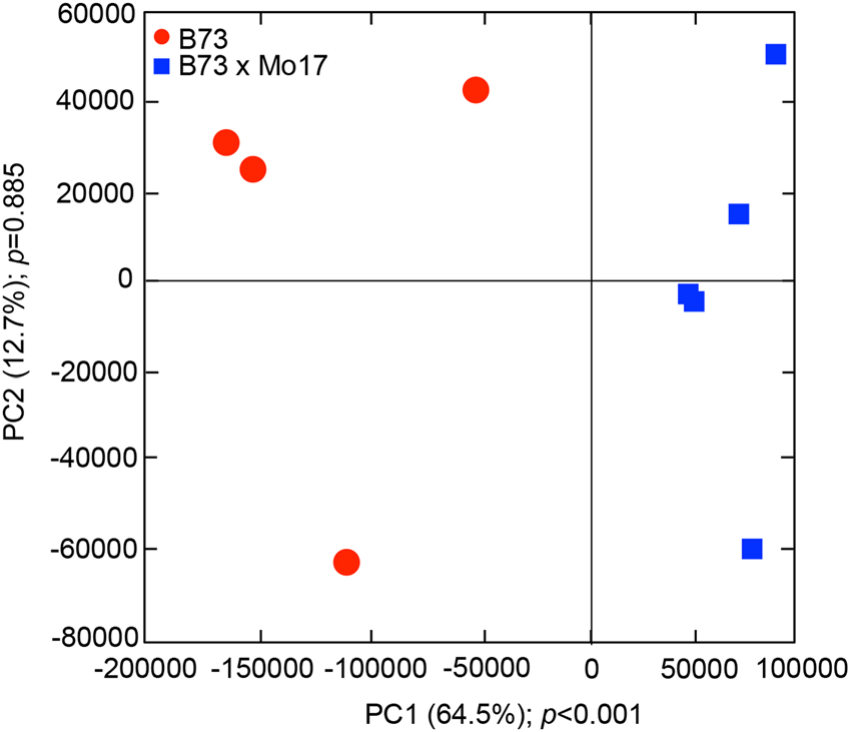
Principal component analysis of the relatedness of root exudates from B73 (n = 4) and B73 x Mo17 (n = 5), which were collected from the 7-day-old seedlings. The first principal component (PC1) explains most of the variation in the data (64.5%) significantly (*p* < 0.001) showing clear separation between genotypes at the first axis. The red circles represent the samples from genotype B73 and blue squares from B73 x Mo17.

To test the hypothesis that the root exudates of the two different maize genotypes shape the microbial communities in the rhizosphere and soil, we conducted a lab-based evaluation of root exudates. Highly significant differences in exudate profiles were found between inbred B73 and hybrid B73 x Mo17 (Fig. 5 and Dataset S4).

### Association of significantly enriched OTUs with eO_3_ in maize

A differential abundance analysis was used to fully understand the differences in OTUs in the B73 endosphere between the aO_3_ and eO_3_ conditions (Fig. S12a,b) and in soil where B73 x Mo17 was grown (Fig. S12c,d). There were 102 OTUs significantly enriched under eO_3_ conditions (Dataset S3) in the B73 endosphere. Of the 102 OTUs 57.8% belonged to the Proteobacteria (Fig. S12b), 21.6% belonged to Actinobacteria, 8.8% belonged to Tenericutes with the rest belonging to Acidobacteria, Bacteroidetes, Firmicutes, Gemmatimonadetes, TM7, and Verrucomicrobia. There were 285 OTUs were depleted under the eO_3_ conditions in B73 endosphere (Dataset 3)  and more than half (51.2%) belonged to Proteobacteria, followed by Actinobacteria (23.9%), Bacteroidetes (8.4%), and Tenericutes (8.1%) with the rest belonging to Acidobacteria, Chloroflexi, Cyanobacteria, Firmicutes, Planctomycetes, Spirochaetes, TM7, and Verrucomicrobia. We found that OTUs in Pseudomonadaceae, Sphingomonadaceae, Rhizobiaceae, Xanthomonadaceae (Proteobacteria), and Chitinophagaceae (Bacteroidetes) were significantly decreased under the eO_3_ conditions (Fig. S12 a,b).

In the differential abundance analysis of soil microbes (Dataset S5) there were 320 OTUs significantly less abundant in the eO_3_ soil where B73 x Mo17 was grown. 21.6% belonged to Proteobacteria, 25% belonged to Actinobacteria, 15% belonged to Acidobacteria, 10.4% belonged to Chloroflexi, 7.8% belonged to Verrucomicrobia and 5.6% belonged to Planctomycetes (Fig. S12c,d). There were also 1158 OTUs significantly enriched in the soil of B73 x Mo17 under eO_3_ conditions (Fig. S12d), as well as many appearances of new phyla (Fig. S12b,d). Among these enriched soil OTUs, 27.6% belonged to the Proteobacteria on the phylum level, 23.7% belonged to Actinobacteria, 15.5% belonged to Acidobacteria, 6.7% belonged to Chloroflexi, 6.4% belonged to Verrucomicrobia and 6.9% belonged to Planctomycetes. Analysis on the family level revealed enrichment of nitrifying bacteria including Nitrososphaeraceae, Nitrospiraceae, Nocardioidaceae, and 0319-6A21 in B73 x Mo17 soil. Simultaneously, the OTUs of N_2_ fixing bacteria including Sphingomonadaceae, Rhizobiaceae, Thermomonosporaceae, Micromonosporaceae, Streptomycetaceae and Bradyrhizobiaceae were also significantly enriched under eO_3_ conditions, suggesting that eO_3_ impacted the nitrogen cycling in the soil of where B73 x Mo17 was grown.

## Discussion

Using culture-independent methods, this study revealed that the species diversity of soil bacterial communities of both maize and soybean was significantly higher than in the endosphere and RHZ (Table 1a). This result is commonly found in the analysis of these different compartments^47,48^. The microbial diversity and richness in the RHZ was greater than in the endosphere, but less than in the soil. Such gradients in microbial diversity between soil and root samples suggest that plants recruit microorganisms from the adjacent soil to construct mutualistic associations^49^. As soybean and maize were grown in the same field, an α-diversity comparison between the two crops, vastly different in their above and belowground growth and reproductive development, was possible. The endosphere and RHZ microbial community diversity was significantly higher in maize. In soybean, the microbial communities were dominated in both elevated and ambient CO_2_ conditions by Rhizobiales (average of 21% relative abundance in RHZ, 75% in endosphere, 7% in soil), which may outcompete other bacterial groups/phyla and lead to a lower diversity of microbial species found in the RHZ and soil of soybean (Table 1b). Such a change in the microbial community, due to the presence or absence of Rhizobia, was recently shown using *Lotus japonicu*s non-nodulating mutants^50^. The β-diversity or community composition was also significantly different between maize and soybean endosphere, RHZ and soil samples indicating that the effect of plant species (even within a single season of growth) has a profound impact on the microbial community composition^51–53^. The significant differences between soybean and maize in both α and β-diversity highlight the distinct host-dependent influence that is emerging as a general principle in plant root – soil microbial interactions in a wide range of ecosystems where the plants play an important role in shaping microbial community structure^47,54^.

The enhanced N use efficiency and productivity of crops under eCO_2_ conditions is well documented^9,55^. Although previous results demonstrate that eCO_2_ alters the soil microbial community function and stimulates the C and N cycling of soil^21,43,44,56^, few studies have characterized the microbial communities under eCO_2_ conditions using 16S amplicon sequencing to determine changes in the microbiome community composition.

In this study, eCO_2_ led to a reduction in the microbial species diversity in the soybean RHZ after a single season, which was not detected in previous studies at this FACE site^43,44^. The RHZ was the only sample type where significant differences between treatments were found under this regime (Fig. 2). This change in the RHZ microbiome community highlights the potential role of exudates in shaping the RHZ. Previous studies have shown that high CO_2_ increases the photosynthetic rate of soybean^55^, which has the potential to increase the rate of C exudation from roots and leading to the observed changes in microbial communities.

Due to the enhanced productivity under eCO_2_^55^ conditions, an increased nutrient supply is required. The diazotrophic rhizobia bacteria involved in interactions with legumes are members of the alpha-subgroup of Proteobacteria. The increase in N_2_ fixing bacteria observed in this study is consistent with the increased demand for N when carbon fixation and yield increases. In this study, the Rhizobiales that fix nitrogen and are symbiotic increased in the endosphere, with a relative abundance of 83% under eCO_2_ conditions compared with 66% in the control. The relative abundance of this same component was 24% in eCO_2_ conditions as compared with 17% in aCO_2_ in RHZ samples. The GeoChip functional data showing an enhancement of soil microbial processes related to N cycling^43,44^ corresponds to these findings. These results are consistent with the significantly decreased species diversity of the RHZ observed under eCO_2_ conditions, indicating the microbial communities were dominated by nitrogen-fixers (Table 1b). Additionally, although the overall microbial communities in the endosphere and soil were not significantly altered by eCO_2_ when analyzed as a population, differential absolute abundance analysis (DESeq. 2) showed that 96 out of 143 OTUs significantly enriched in the endosphere under eCO_2_ conditions belonged to N_2_ fixing bacteria, including unclassified members of Rhizobiales and Burkholderiaceae. This finding also corresponds to previous results showing that eCO_2_ stimulated increases in both the mass and number of soybean nodules, along with a 33% increase in shoot C and a 78% increase in N uptake^57^.

As tropospheric O_3_ is one of the most widespread phytotoxic air pollutants^58^, it is important to understand the effect of O_3_ on below-ground processes, mediated by the soil microbial community. Significant treatment differences were found in the analyses of each maize genotype, showing that microbial community composition shifts due to eO_3_. One of these differences was found in inbred B73 endosphere samples (UUF analysis only) and the second difference was observed in the soil where B73 x Mo17 was grown (both WUF and UUF analyses). However, this change was not observed in the soil where B73 was grown, indicating differential genotypic responses to eO_3_ conditions. A previous study of eO_3_ found small RHZ bacterial community diversity changes in both O_3_-tolerant and sensitive herbaceous plants, despite leaf injury and chlorosis on sensitive plants^59^. Two other studies on wheat^60^ and rice^61^ also showed decreased diversity and reduced biomass due to eO_3_. In contrast to our findings, the study on wheat^60^ only reported reduced changes in diversity and microbial biomass in the RHZ and did not measure the microbial compositional changes with high resolution 16S amplicon sequencing. In yet another study on rice, no statistically signficant differences were found in eO_3_ treatments^62^. We speculate that the soil effects seen where the hybrid maize was grown may be due to the enhanced stress tolerance of hybrids and root exudation, whereas the changes observed in the endosphere may be due to the higher stress the inbred maize may be experiencing. As changes in the soil microbiome under eO_3_ conditions were only observed in the soil where B73 x Mo17 was grown, these are unlikely the result of direct O_3_ diffusion into the soil^59,63,64^, but rather from plant-specific factors.

The endosphere, RHZ and soil samples all showed strong statistically significant genotypic differences in microbial community composition. The differential abundance analysis in the soil where the hybrid B73 x Mo17 was grown showed that many nitrifying and N_2_ fixing bacteria were enriched in eO_3_, indicating that eO_3_ had an impact on N cycling processes. Nitrifying bacteria, including Nitrososphaeraceae, Nitrospiraceae, Nocardioidaceae, and 0319-6A21^65–67^, were significantly enriched under eO_3_ conditions in B73 x Mo17 soil. In contrast to the maize hybrid, a previous study of soybean grown under eO_3_ reported an overall decrease in the abundance of N cycling genes, but an increase in soil nitrate between 5–15 cm depth in the soil^43^. This finding indicated that nitrifying bacteria may be enriched in eO_3_ conditions due to a reduction in nitrogen uptake, allowing nitrifying bacteria a greater opportunity to compete for N.

In this study, we found significant differences in microbial community composition due to the maize genotype in the aO_3_ and eO_3_ conditions across all the sample types (Figs 3, 4, S7, 8, 9, 11c,e,f). The significant differences in microbial communities between two genotypes of the same plant species (*Zea mays*) supports the hypothesis that the host shapes the root microbiome. Root exudates from plants^68–71^ and compounds sloughed off root tips^47^ are important in shaping the microbial communities of the rhizosphere and endosphere. Root exudates may shape microbial communities due to the sugars, amino acids, organic acids and hormones that they contain^72^. Root exudates are also important due to the “rhizosphere” effect, where the density of bacteria is high in the RHZ due to the enhanced availability of carbon exuded by roots^68^. To test the hypothesis that differences in the composition of root exudates influence microbial community composition, B73 and B73 x Mo17 exudates were compared by GC-MS using multivariate analysis. The results showed that exudates were clearly distinct in the inbred and hybrid lines. Future work will focus on further characterizing the compounds that differed between the two lines. Genotypic differences in *Carex arenaria* and *Festuca rubra* root exudates under eCO_2_ conditions have been studied to a limited extent. The different root exudate compositions between the two species led to changes in the abundance of selected members of the bacterial and fungal microbiome in that system^73^. In the endosphere, where microbes have direct contact with the root apoplast, additional factors may limit and control microbial abundance, including well-characterized, host-specific factors^74,75^. These host-specific factors may account for the differences in the maize endosphere between the inbred and hybrid varieties. The differences in root exudates is exciting and may lead to more applied biological engineering projects designed to use root exudates to create specific environments that encourage the growth of the most beneficial microbes around roots.

The rhizosphere, endosphere and bulk soil are comprised of multiple physicochemical factors and biological organisms such as bacteria, fungi, nematodes, worms and other organisms. The microbial communities characterized in this study are complex and dynamic with respect to root proximity, plant response to global climate change factors as well as plant species and genotype. The changes measured in this study occurred rapidly, in one season of growth, and indicate that soil microbial communities in proximity to plant roots will change with the shift of global climate toward higher concentrations of atmospheric gases, such as O_3_ and CO_2_.

## Methods

### Study site

We conducted this study during the summer of 2014 at the Free Air Concentration Enrichment (FACE, http://soyface.illinois.edu/) facility in Champaign, Illinois, USA (40°03′21.3″N, 88°12′3.4″ W, 228 m elevation). Octagonal plots (20 m dia) were enriched with CO_2_ to a target concentration of 600 ppm or O_3_ to a target concentration of 100 ppb by fumigation^6,76^. In the field there were eight blocks for the elevated O_3_ treatment and controls and four blocks for the elevated CO_2_ treatment and controls (Fig. S13). The site was comprised of Drummer silty clay loam and Flanagan silt loam (Fig. S13)^77^. Soil type was included in the CAP analysis as a random variable, given that it can significantly affect the microbial communities (Fig. S5). Soil chemical analysis was performed by Ward Labs, Kearney, Nebraska (Dataset S1). The maize inbred B73 and the hybrid B73 x Mo17 were grown in aO_3_ and eO_3_ (n = 4). The Pioneer 93B15 soybean variety was grown in aCO_2_ and eCO_2_ conditions (n = 4). Maize seed was produced in nurseries on the University of Illinois, Champaign south farms and soybean seed was provided by Pioneer.  Field plots were directly seeded. Maize plants were at the R2 (reproductive) developmental stage or the blister stage and one plant per plot was excavated to approximately 16–20 cm. The soybean plants were between the R5–R6 or seed filling stage and two samples were taken per plot, each containing 2–4 plants. Only lateral roots were collected from soybean roots and nodules were removed from the soybean roots in the field. Roots were separated from the plant and placed in 50 ml tubes containing phosphate buffer. Tubes were shaken for 1 min and roots were removed, blotted dry and placed into a clean tube. The phosphate buffer containing rhizosphere soil shaken off from roots and the roots were placed on ice. A sample of soil was collected from about 0 – 16 cm from the roots and kept on ice until processing. Roots were sterilized with 5.25% sodium hypochlorite and 70% ethanol, frozen and then ground with liquid nitrogen. The RHZ samples were filtered with 100 µm filters, spun down and then frozen. Root pieces were removed from soil and soil was stored at 4 °C until DNA extraction. Soil was sieved before chemical analysis and sieves were cleaned thoroughly between each sample.

### DNA extraction and 16S rRNA gene sequencing

DNA was extracted from soil and rhizosphere using the PowerSoil-htp 96 Well Soil DNA Isolation Kit (Catalog No. 12955-4, MoBio, Carlsbad, CA, USA). Root DNA was extracted with the PowerPlant Pro-htp 96 Well DNA Isolation Kit (Catalog No. 13496-4, MoBio, Carlsbad, CA, USA). To characterize the sample microbial populations, the ribosomal primers 515 f and 806r were used to amplify the V4 region of the 16S rRNA gene at the University of Minnesota Genomics Facility using their published protocol^78^. The protocol is included in the Supporting Information. Sequencing was performed using paired-end 250 base reads on an Illumina MiSeq sequencing platform. For root samples, a Illumina HiSeq sequencing platform was used to survey the microbe communities to ensure full coverage of the root diversity because PNA blockers do not fully block the abundant chloroplast and mitocondrial sequences. The same amplification protocols were used, but samples were sequenced using a 250 bp read kit with PNA blockers^79^ to reduce the amount of chloroplast and mitochondrial 16S amplification from roots.

### Sequencing data processing and data analysis

Raw fastq files were de-multiplexed using QIIME (Quantitative Insights into Microbial Ecology, version 1.9.1)^80^ and quality-filtered using trim_galore/0.4 and cutadapt/1.4 (Babraham Bioinformatics). The 250 bp reads were trimmed when the average Phred quality score was less than 20 using default parameters over a 10 bp sliding window. Software pandaseq/2.9 was used to merge the two reads. Operational taxonomic units (OTUs) were determined using an open-reference picking method. A threshold of 97% identify was used to identify OTUs that were grouped together as the same. OTUs were then identified using the Greengenes database (v13_8) by UCLUST. Alignments to determine phylogenetic relationships were done using PyNAST. A total of 20,272,914 and 7,665,572 high-quality sequences were obtained from the maize and soybean samples, respectively. The high-quality sequences were chimera-checked and clustered into 90,884 and 61,881 OTUs in maize and soybean samples, respectively. A phylogenetic tree was built using FastTree and metrics in Newick format were used for both α−and β-diversity analysis. The default settings for building the tree were “-nt -gamma -fastest -no2nd -spr 4” configured in OTU picking and phylogenetic tree building scripts in QIIME. Taxonomy was subsequently assigned to each representative OTU using the Ribosomal Database Project (RDP) classifier with a standard minimum support threshold of 80%. Sequences identified as chloroplasts or mitochondria were removed from the analysis. Low abundance OTUs (<5 total counts) were discarded, resulting in 34,602 and 18,084 OTUs in maize and soybean samples, respectively. To analyze all data together, (both sample type and crop species), we rarefied to 1901 (Fig. S3) and 1405 (Fig. S7) sequences per sample in soybean and maize samples, respectively, to correct for differences in reads across samples. When the different sample types were analyzed as different communities, we rarefied to 1891 sequences per sample for endosphere, 9264 for rhizosphere, and 4232 for soil in soybean, and 1605 sequences per sample for endosphere, 5145 for rhizosphere, and 4971 soils in maize (one root sample was discarded due to an extremely low sequencing depth). Community alpha diversity was estimated using the Shannon diversity index in QIIME^81^. Both WUF and UUF distance matrices were used to perform unconstrained principal coordinate analysis (PCoAs) and Canonical Analysis of Principal coordinates (CAP) analysis^82,83^. In these analyses, we constrained the analysis to the factor of interest while controlling for all other experimental factors (i.e., block and soil type) and technical factors (sequencing method^35^) by using the function capscale in the vegan package^84^. PCoA data are shown, but similar results were found in a comparison with non-metric multi-dimensional scaling (NMDS) using the UniFrac matrix. In addition, eigenvalues for PCoA were all positive, except for a few negative values (small magnitude) that were replaced with zero during the analysis, indicating that non-Euclideanarity was not a problem and data transformation was not necessary^85^. Analysis of variance (PERMANOVA) was performed to determine statistical significance. Differentially abundant OTUs were identified using DESeq. 2 in QIIME^86^. The significant changes in OTUs were visualized using Phyloseq.^87^ and iTOL^88^.

The linear discriminant analysis (LDA) effect size (LEfSe) algorithm (https://huttenhower.sph.harvard.edu) was used to identify significant differences in microbial communities based on different taxonomic soybean ranks. An α-value for the factorial Kruskal-Wallis test was set at 0.05 and threshold for the logarithmic LDA score was 3.0.

### Preparation of root exudate samples

B73 and B73 X Mo17 maize seeds were sterilized with 70% ethanol for two minutes and 50% bleach for ten minutes and put on germination paper. After seven days of growth, maize seedlings were removed from paper. Complete root systems of seedlings from 10 plants were pooled together and inserted into a 50 ml tube filled with 35 ml of ultrapure water with a low concentration of CaCl_2_ (2 mmol) to collect the root exudates. After 2 h, the solution in which roots were placed was filtered (0.22 μm pore size) to remove root debris and microbial cells. The filtered samples were kept frozen at −80 °C until they were concentrated. The fresh roots were cut, blotted dry and then weighed. The root exudates were lyophilized to remove water, resuspended in 1 ml extraction buffer (2% acetic acid in methanol) and sonicated for 5 min to improve the dissolution. The extracts were then centrifuged for 10 min at 12,600 × g and stored at −80 °C before GC-MS analysis.

### GC-MS analysis of root exudates

Samples were re-suspended in a 1:20 (v/v) methanol solution and an aliquot was dried under N_2_. The samples were re-suspended in a pyridine solution containing 50 mg/ml of methoxyamine hydrochloride, incubated at 60 °C for 45 min, sonicated for 10 min and incubated at 60 °C for an additional 45 min. Next, 50 μl of N-methyl-N-trimethylsilyltrifluoroacetamide with 1% trimethylchlorosilane (MSTFA + 1% TMCS, Thermo Scientific) was added and samples were incubated at 60 °C for 30 min, cooled to room temperature and the supernatant was transferred to a GC-MS autosampler vial. Metabolites were detected using a Trace 1310 GC coupled to a Thermo ISQ-LT MS (Thermo Scientific). For separation, a 30 m TG-5MS column (Thermo Scientific, 0.25 mm internal diameter, 0.25 μm film thickness) with a 1.2 ml/min helium gas flow rate was used. QC samples pooled from the set of samples were derivatized in the same manner described above and injected after every two samples.

## Electronic supplementary material

Supplementary Figures

Datasets 1,2,3,4
