# Supplementary material for: Shifts in microbial communities in soil, rhizosphere and roots of two major crop systems under elevated CO2 and O3
Source: Sci Rep. 2017 Nov 3;7:15019. doi: 10.1038/s41598-017-14936-2 (PMC5670137; doi:10.1038/s41598-017-14936-2)
Supplement: Supplementary file 1 — Supplementary Figures [file 41598_2017_14936_MOESM1_ESM.pdf]

Shifts in microbial communities in soil, rhizosphere and roots of two major cropping systems under elevated CO_2 and O_3

Peng Wang[1], Ellen L. Marsh[1], Elizabeth A. Ainsworth[2,3], Andrew D.B. Leakey[2], Amy M. Sheflin[4], Daniel P. Schachtman[1*]

[1]Department of Agronomy and Horticulture, University of Nebraska Lincoln, Lincoln, NE 68588, USA.

[2]Department of Plant Biology, University of Illinois at Urbana-Champaign, Urbana, IL 61801, USA.

[3]USDA ARS Global Change and Photosynthesis Research Unit, Urbana, IL 61801, USA.

[4]Proteomics and Metabolomics Facility, Colorado State University, Fort Collins, CO, 80523, USA

*Author for correspondence:

Daniel Schachtman

Tel: +1- 314 799 2427 Email: daniel.schachtman@unl.edu

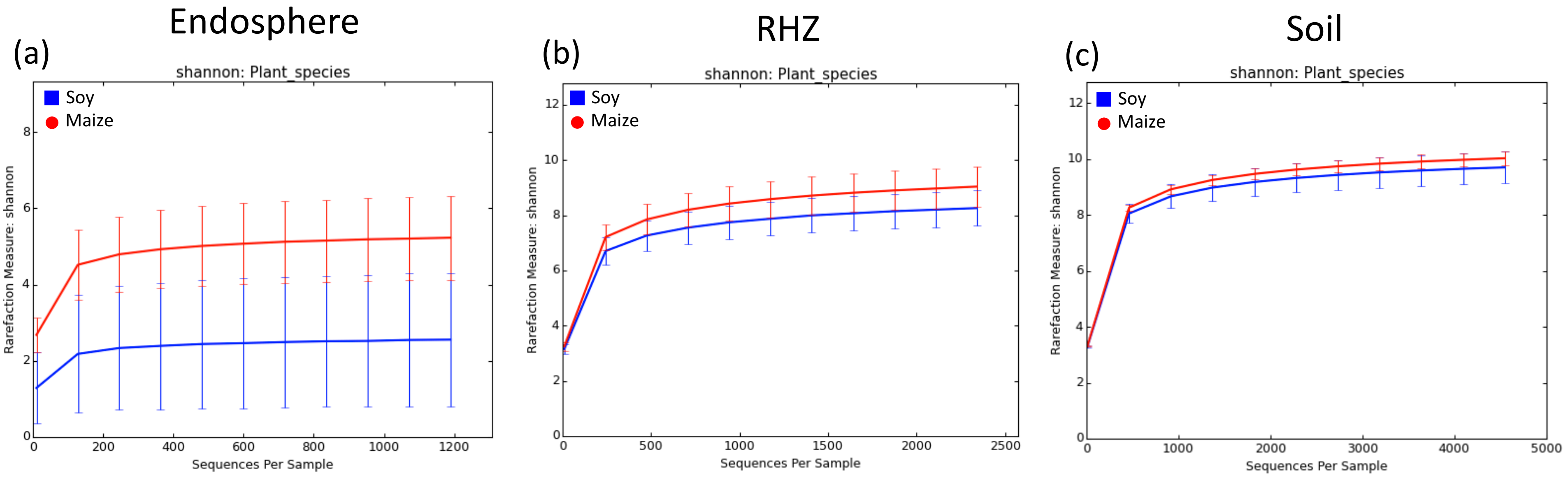

Fig. S1 Rarefaction curve of endosphere, RHZ, and soil of soy and maize.

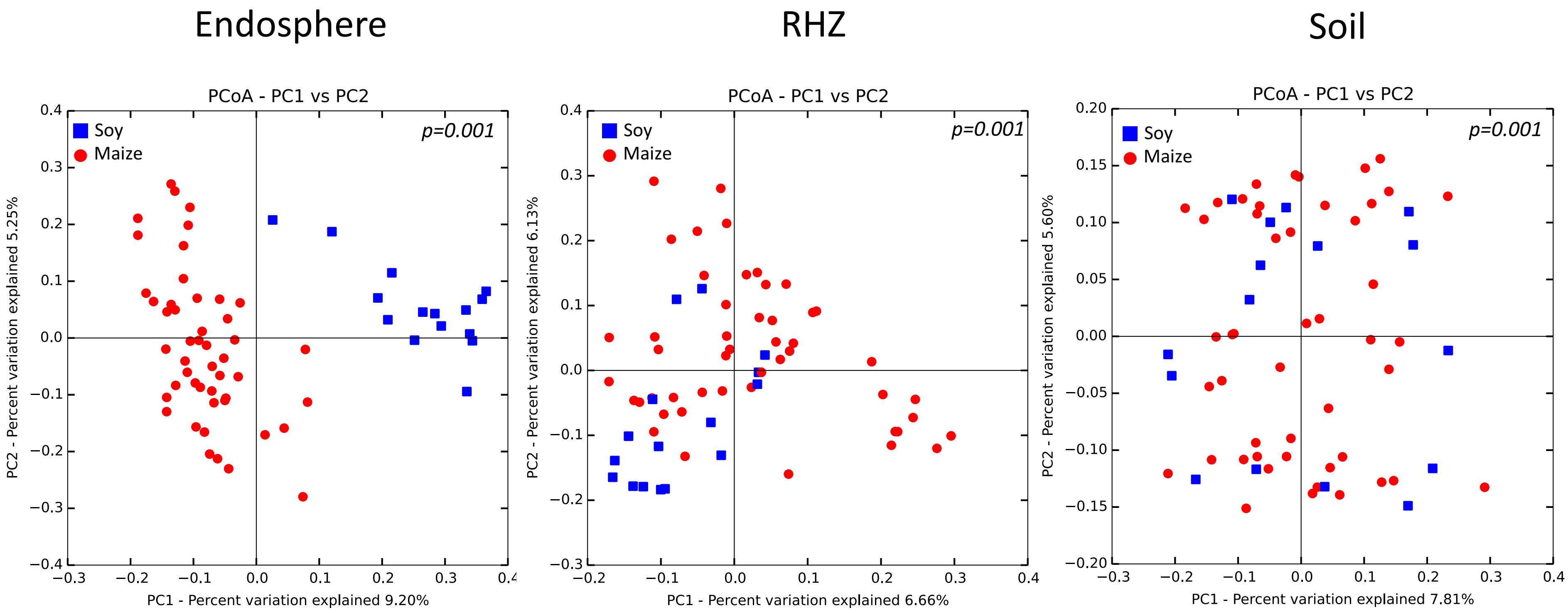

Fig. S2 Unweighted UniFrac distance matrix (UUF) shows significant difference between plant species in microbial taxa under ambient conditions.

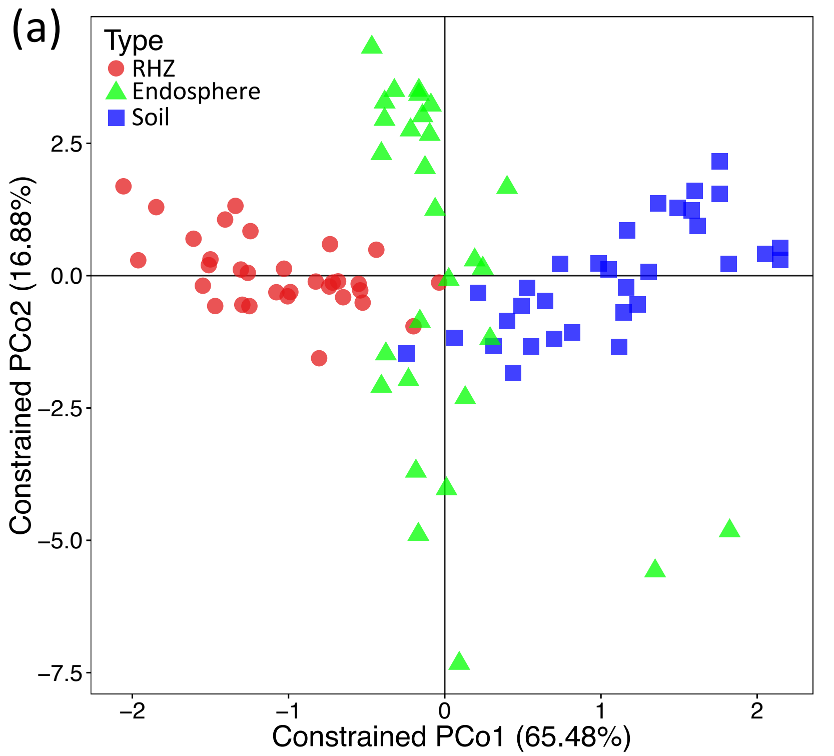

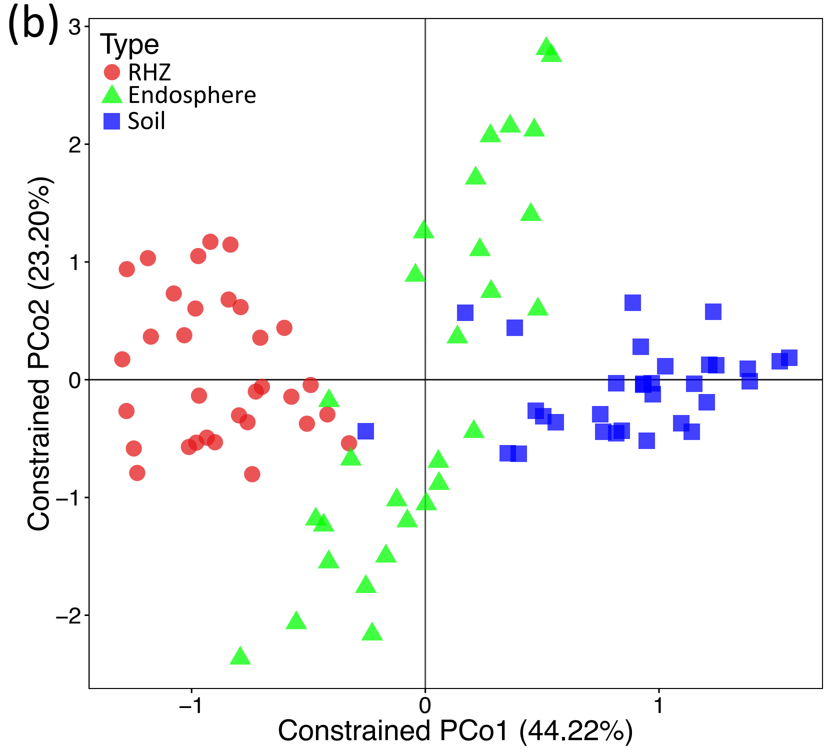

Model: capscale(formula = as.dist(gh.wuf) ~ Type * Treatment + Condition(SoilType + Sequencing), data = gh.map, add = T)

Factor	Df	SumOfSqs	F	Pr(>F)
Type	1	2.90	7.70	0.001 ***
Treatment	1	0.45	1.18	0.20
Type:Treatment	2	1.19	1.58	0.009 **
Residual	86	32.43		

Signif. codes: ‘***’: ≤0.001, ‘**’: ≤0.01, ‘*’: ≤0.05, ‘.’: ≤0.1, ‘ ’: >0.1

Model: capscale(formula = as.dist(gh.uuf) ~ Type * Treatment + Condition(SoilType + Sequencing), data = gh.map, add = T)

Factor	Df	SumOfSqs	F	Pr(>F)
Type	1	0.70	2.93	0.001 ***
Treatment	1	0.24	0.99	0.47
Type:Treatment	2	0.68	1.41	0.001 ***
Residual	86	20.61		

Signif. codes: ‘***’: ≤0.001, ‘**’: ≤0.01, ‘*’: ≤0.05, ‘.’: ≤0.1, ‘ ’: >0.1

Fig. S3 CAP analysis using WUF (a) and UUF (b) matrix in soy constrained to sample type and treatment and controlling for soil type and sequencing method. PERMANOVA result indicated that different sample types have a significant effect on microbial community composition.

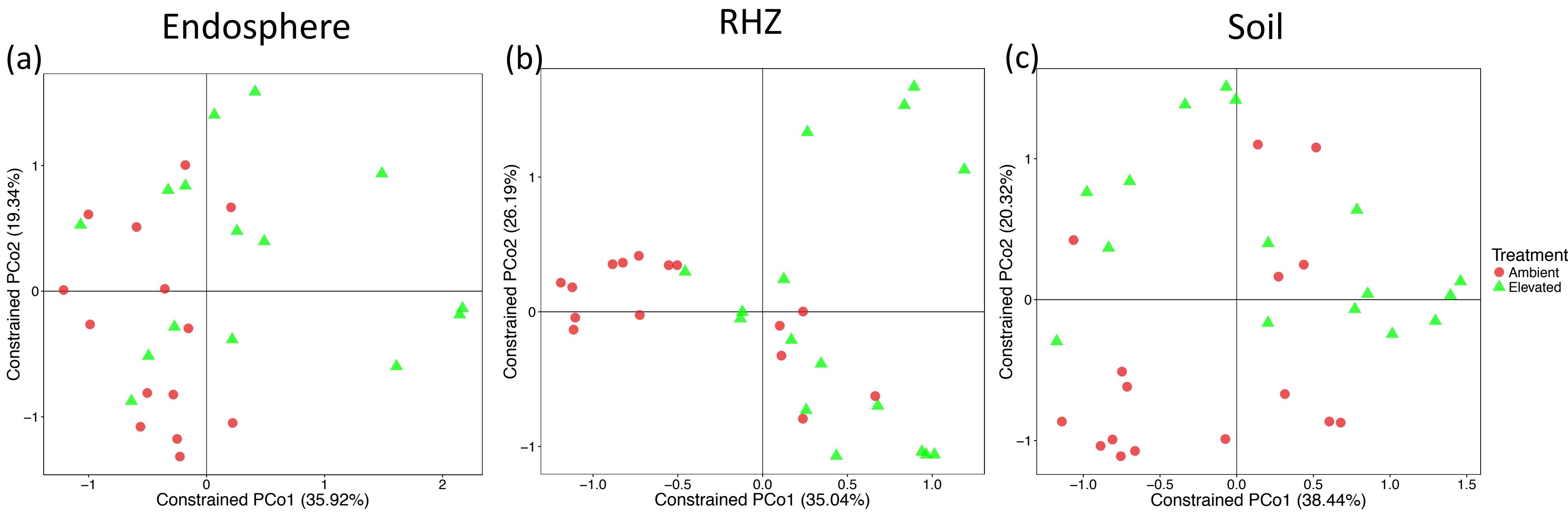

Model: capscale(formula = as.dist(gh.uuf) ~ Treatment + Block + SoilType + Condition(Ring), data = gh.map, add = T)

Factor	Df	SumOfSqs	F	Pr(>F)
Treatment	1	0.34	1.29	0.095 .
Block	3	0.86	1.08	0.27
SoilType	1	0.28	1.06	0.33
Residual	22	5.90		

Signif. codes: 0 ‘***’ 0.001 ‘**’ 0.01 ‘*’ 0.05 ‘.’ 0.1 ‘ ’ 1

Model: capscale(formula = as.dist(gh.uuf) ~ Treatment + Block + SoilType + Condition(Ring), data = gh.map, add = T)

Factor	Df	SumOfSqs	F	Pr(>F)
Treatment	1	0.28	1.98	0.003 **
Block	3	0.55	1.29	0.02 *
SoilType	1	0.21	1.51	0.02 *
Residual	24	3.45		

Signif. codes: 0 ‘***’ 0.001 ‘**’ 0.01 ‘*’ 0.05 ‘.’ 0.1 ‘ ’ 1

Model: capscale(formula = as.dist(gh.uuf) ~ Treatment + Block + SoilType + Condition(Ring), data = gh.map, add = T)

Factor	Df	SumOfSqs	F	Pr(>F)
Treatment	1	0.21	1.12	0.23
Block	3	0.63	1.09	0.20
SoilType	1	0.28	1.46	0.03 *
Residual	25	4.85		

Signif. codes: 0 ‘***’ 0.001 ‘**’ 0.01 ‘*’ 0.05 ‘.’ 0.1 ‘ ’ 1

Fig. S4 Factors influencing endosphere, RHZ and soil microbiome β-diversity in soy analyzed using UUF matrix. CAP analysis shown.

(a)

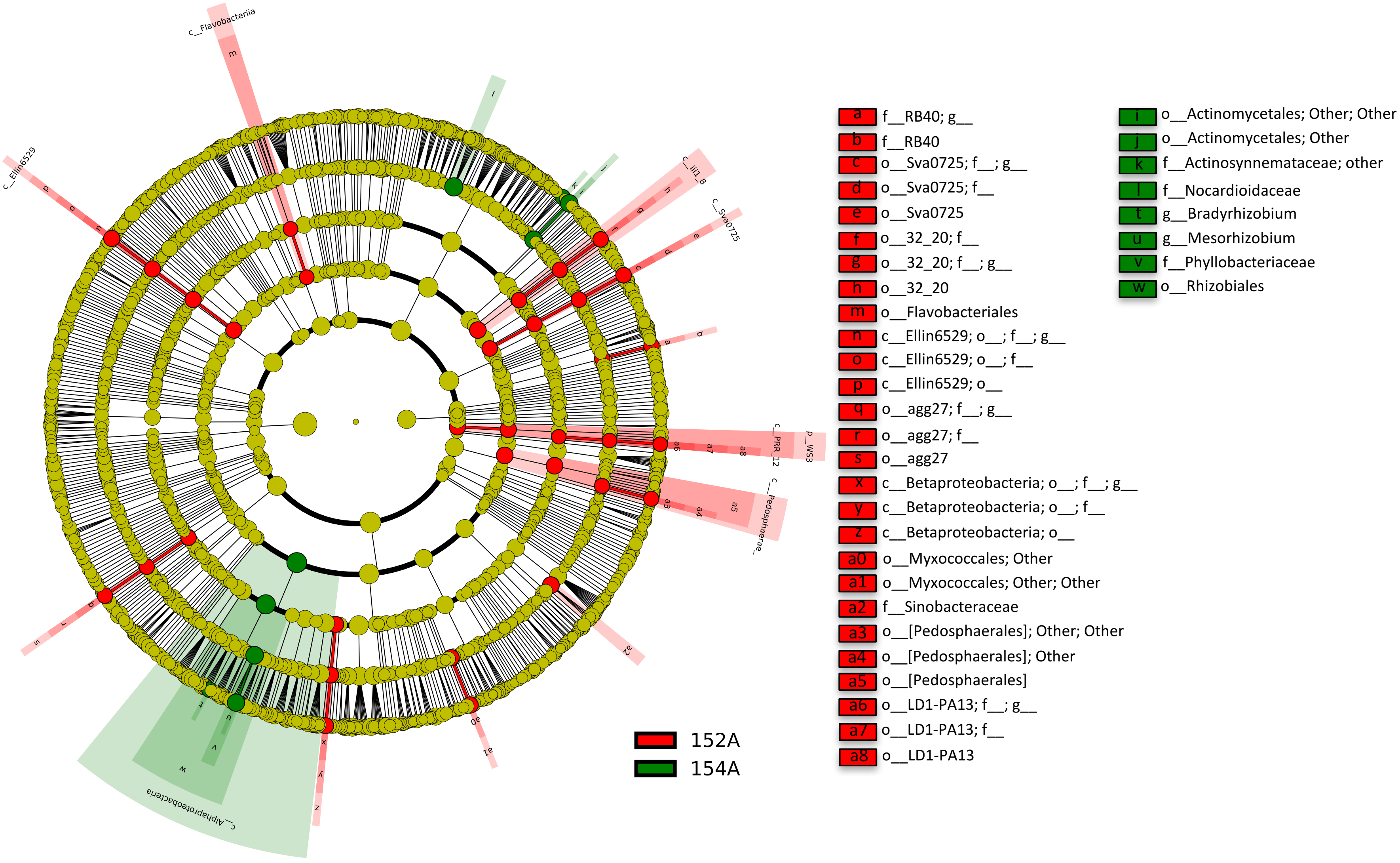

Fig. S5 Relative abundance of microbial communities of different taxonomic rank between two soil types 152A and 154A in soil of soy field. 152A: Drummer silty clay loam, 0 to 2 percent slopes; 154A: Flanagan silt loam, 0 to 2 percent slopes. Nitrogen cycle related bacteria are enriched in the 154A soil type (green) as compared to 152A soil type.

Fig. S5 Continued

(b)

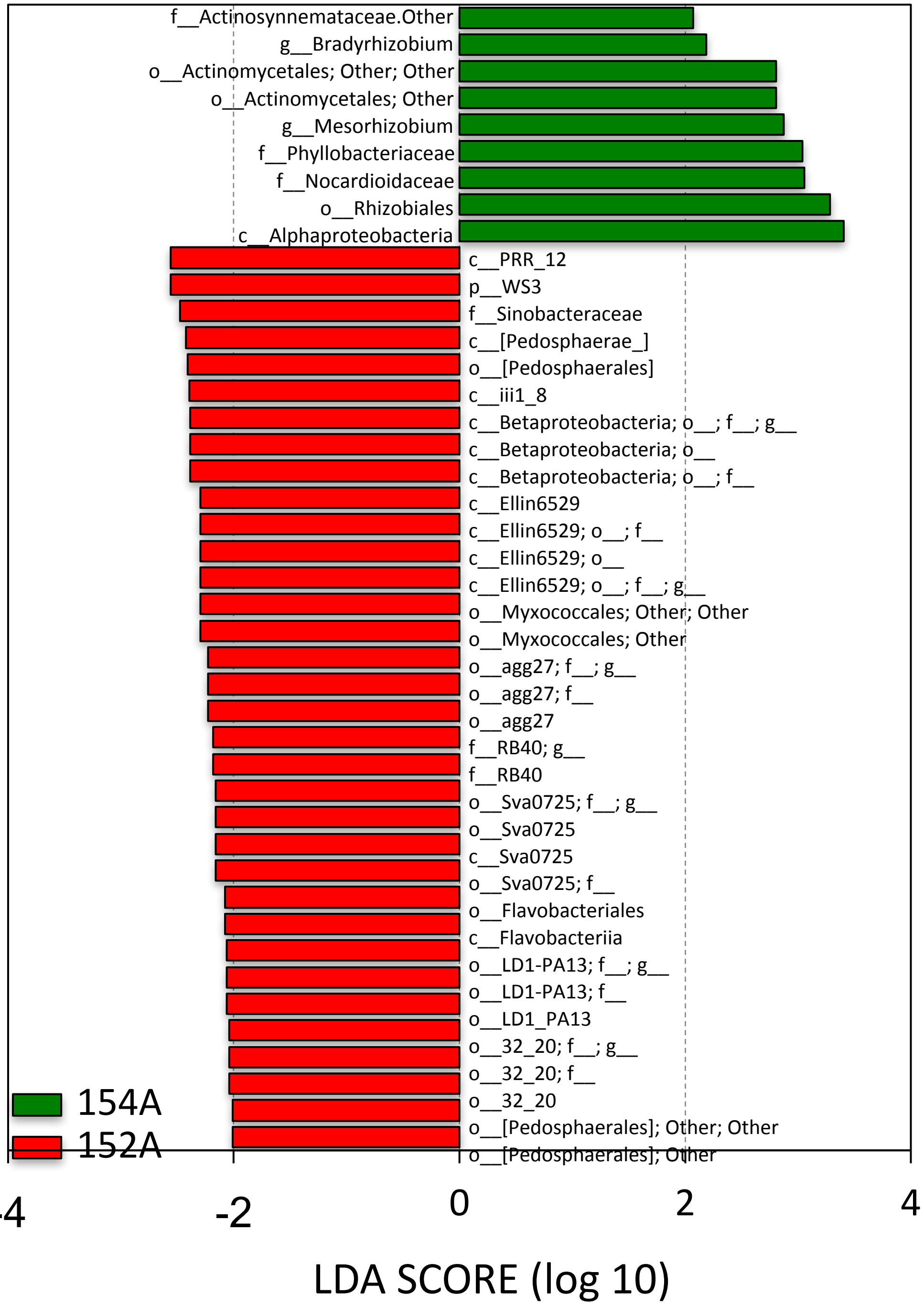

Fig. S5 Differential relative abundance of microbial communities on two soil types 152A and 154A in soil of soybean field. 152A: Drummer silty clay loam, 0 to 2 percent slopes; 154A: Flanagan silt loam, 0 to 2 percent slopes. Nitrogen cycle related bacteria are enriched in the 154A soil type (green) and more than 152A soil type.

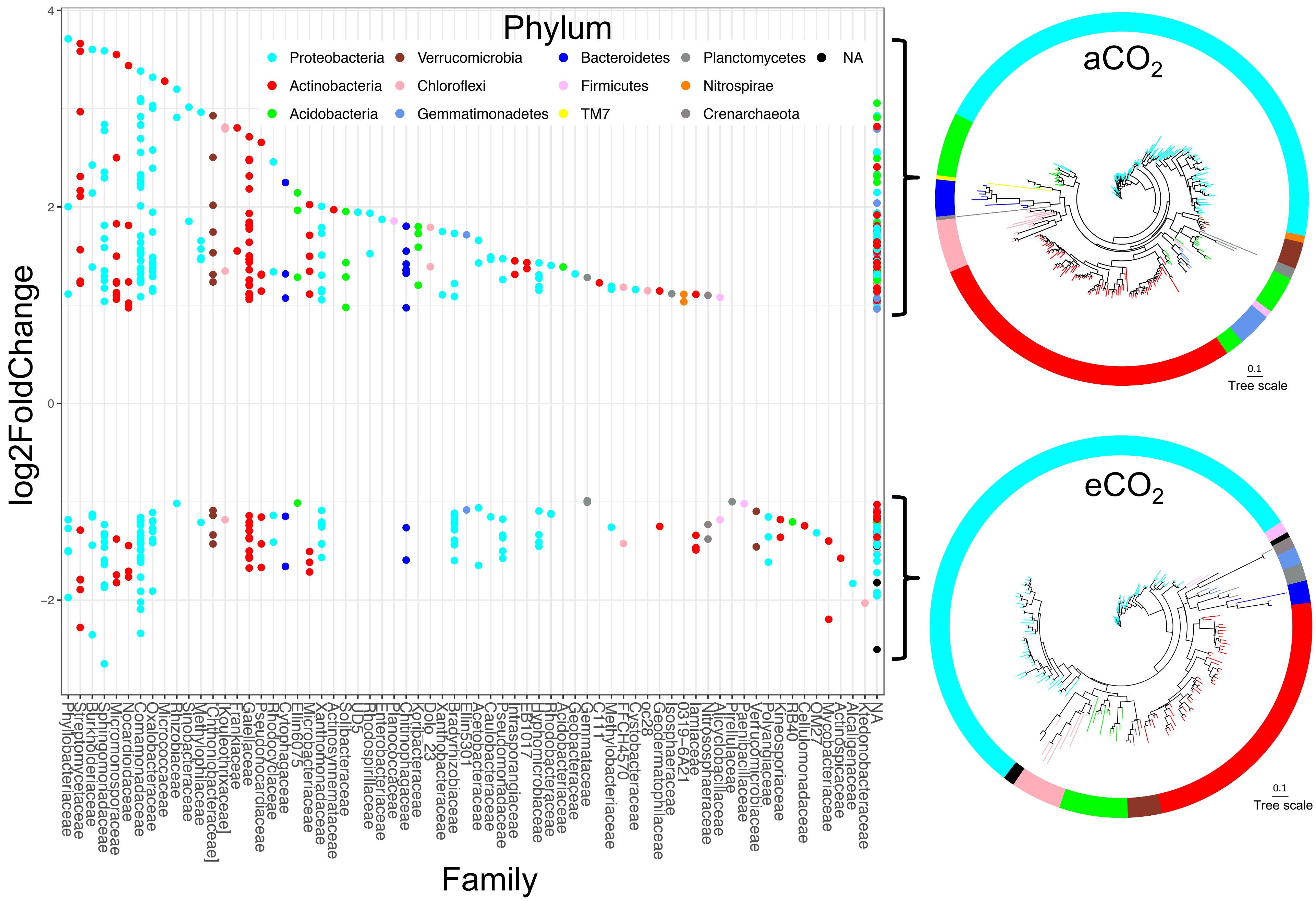

Phylum
Proteobacteria
Verrucomicrobia
Bacteroidetes
Planctomycetes
NA
Actinobacteria
Chloroflexi
Firmicutes
Nitrospirae
Acidobacteria
Gemmatimonadetes
TM7
Crenarchaeota
log2FoldChange
4
2
0
−2
Family
Phyllobacteriaceae
Streptomycetaceae
Burkholderiaceae
Sphingomonadaceae
Micromonosporaceae
Nocardioidaceae
Comamonadaceae
Oxalobacteraceae
Micrococcaceae
Rhizobiaceae
Sinobacteraceae
Methylophilaceae
[Chthoniobacteraceae]
[Kouleothrixaceae]
Frankiaceae
Gaiellaceae
Pseudonocardiaceae
Rhodocyclaceae
Cytophagaceae
Ellin6075
Microbacteriaceae
Xanthomonadaceae
Actinosynnemataceae
Solibacteraceae
UD5
Rhodospirillaceae
Enterobacteriaceae
Planococcaceae
Chitinophagaceae
Koribacteraceae
Dolo_23
Xanthobacteraceae
Bradyrhizobiaceae
Ellin5301
Acetobacteraceae
Caulobacteraceae
Pseudomonadaceae
Intrasporangiaceae
EB1017
Hyphomicrobiaceae
Rhodobacteraceae
Acidobacteriaceae
Geobacteraceae
Gemmataceae
C111
Methylobacteriaceae
FFCH4570
Cystobacteraceae
oc28
Geodermatophilaceae
Isosphaeraceae
0319−6A21
Iamiaceae
Nitrososphaeraceae
Alicyclobacillaceae
Pirellulaceae
Paenibacillaceae
Verrucomicrobiaceae
Polyangiaceae
Kineosporiaceae
RB40
Cellulomonadaceae
OM27
Mycobacteriaceae
Actinospicaceae
Alcaligenaceae
Ktedonobacteraceae
NA
aCO2
0.1
Tree scale
eCO2
0.1
Tree scale

Fig. S6

Fig. S6 Differential abundance analysis of RHZ of soy. (a) OTUs whose abundance was significantly influenced by elevated CO_2 in RHZ. Each dot in the graph indicates one OTU, which was significantly altered by elevated CO_2 (FDR adjusted $P<0.05$). All the OTUs belonging to the same family (shown at bottom) are in one column. Different colors indicates the different phylum, and the families in the same colour demonstrate that are affiliated with specific phylum. (b) The percentage of the differentially abundant phylum differentially expressed in either ambient or elevated CO_2. The branches inside the circle with the same color indicate that the OTUs belong to same phylum. The phylogenetic tree was generated by using the OTUs that were differentially abundant. The tree scale at the bottom right shows the length of branch that represents the amount of genetic change.

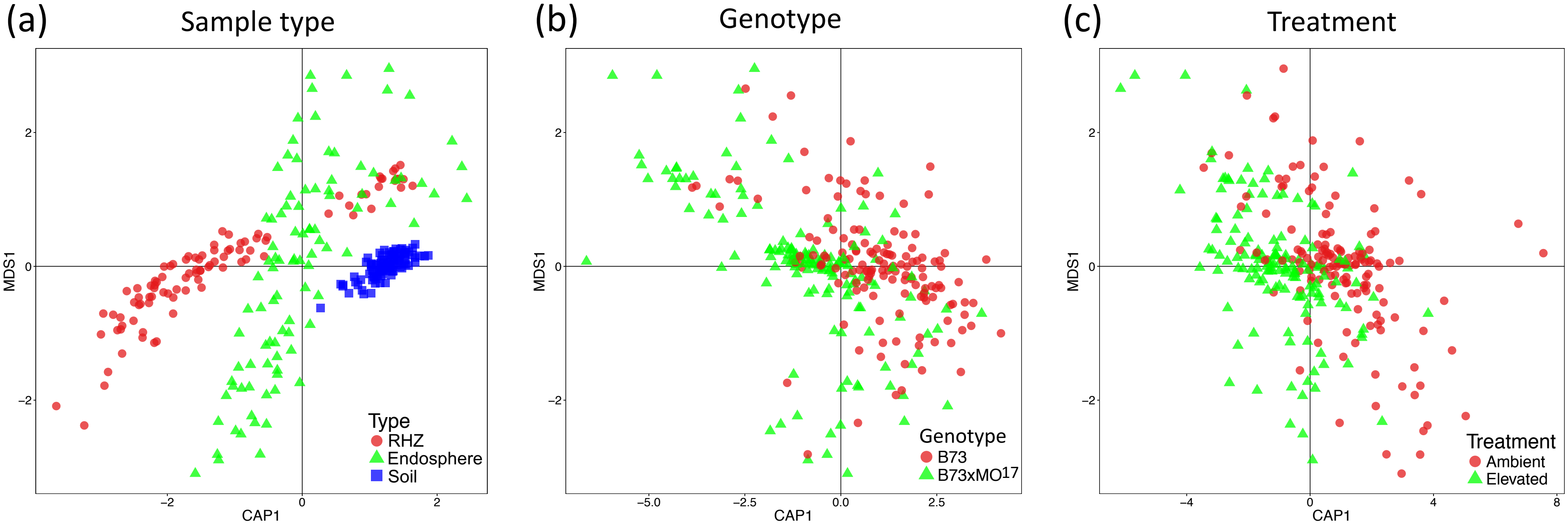

(a) Model: capscale(formula = as.dist(gh.wuf) ~ Type + Condition(SoilType + Treatment + Genotype + Block + Ring + Sequencing), data = gh.map, add = T)

Factor	Df	SumOfSqs	F	Pr(>F)	
Type	1	5.80	8.91	0.001	***
Residual	269	178.95			

Signif. codes: ‘***’: ≤0.001, ‘**’: ≤0.01, ‘*’: ≤0.05, ‘.’: ≤0.1, ‘ ’: >0.1

(b) Model: capscale(formula = as.dist(gh.wuf) ~ Genotype + Condition(SoilType + Treatment + Type + Sequencing), data = gh.map, add = T)

Factor	Df	SumOfSqs	F	Pr(>F)	
Genotype	1	1.14	1.74	0.001	***
Residual	281	184.19			

Signif. codes: ‘***’: ≤0.001, ‘**’: ≤0.01, ‘*’: ≤0.05, ‘.’: ≤0.1, ‘ ’: >0.1

(c) Model: capscale(formula = as.dist(gh.wuf) ~ Treatment + Condition(SoilType + Type + Genotype+ Block + Sequencing), data = gh.map, add = T)

Factor	Df	SumOfSqs	F	Pr(>F)
Treatment	1	0.63	0.96	0.53
Residual	275	178.95		

Signif. codes: ‘***’: ≤0.001, ‘**’: ≤0.01, ‘*’: ≤0.05, ‘.’: ≤0.1, ‘ ’: >0.1

Fig. S7 Ordination of CAP analysis using WUF matrix constrained by sample type, genotype, and treatment in maize.

Fig. S7 continued

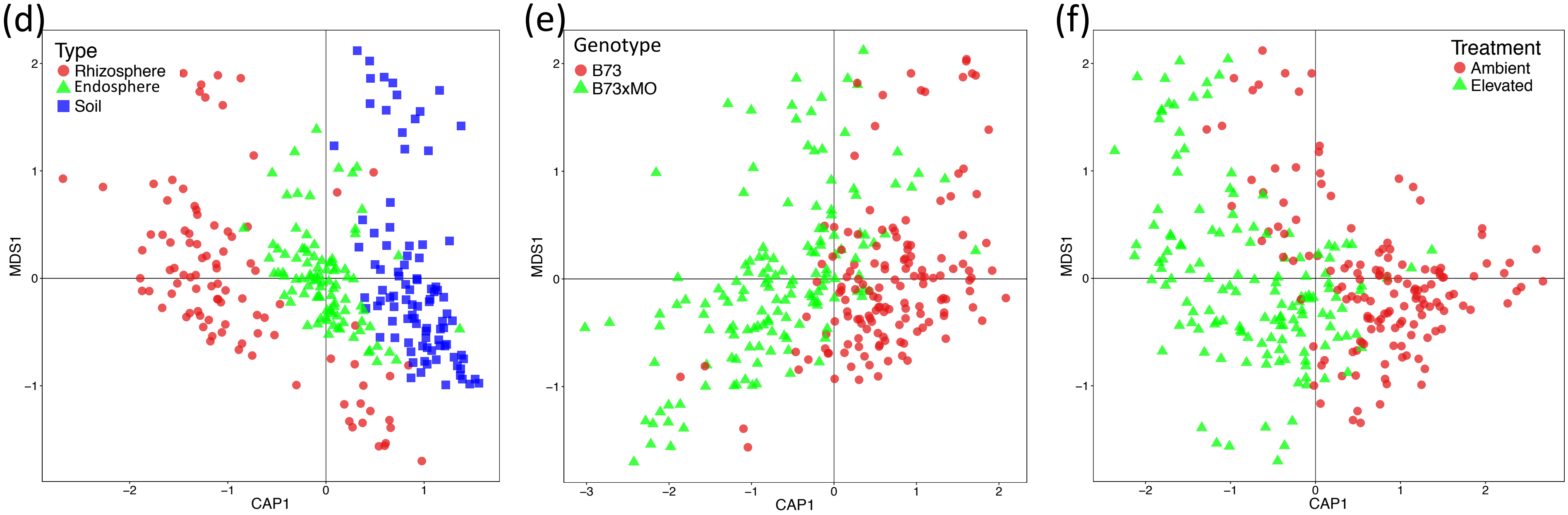

Model: capscale(formula = as.dist(gh.uuf) ~ Type + Condition(SoilType + Treatment +Genotype+ Block + Ring+ Sequencing), data = gh.map, add = T)

Factor	Df	SumOfSqs	F	Pr(>F)
Type	1	1.30	5.39	0.001 ***
Residual	269	66.33		

Signif. codes: 0 '***' 0.001 '**' 0.01 '*' 0.05 '.' 0.1 ' ' 1

Model: capscale(formula = as.dist(gh.uuf) ~ Genotype + Condition(SoilType + Treatment + Type+Sequencing), data = gh.map, add = T)

Factor	Df	SumOfSqs	F	Pr(>F)
Genotype	1	0.37	1.53	0.001***
Residual	281	68.41		

Signif. codes: 0 '***' 0.001 '**' 0.01 '*' 0.05 '.' 0.1 ' ' 1

Model: capscale(formula = as.dist(gh.uuf) ~ Treatment + Condition(SoilType + Type +Genotype+ Block + Sequencing), data = gh.map, add = T)

Factor	Df	SumOfSqs	F	Pr(>F)
Treatment	1	0.30	1.23	0.019*
Residual	275	66.33		

Signif. codes: 0 '***' 0.001 '**' 0.01 '*' 0.05 '.' 0.1 ' ' 1

Fig. S7 Ordination of CAP analysis using UUF matrix constrained by sample type, genotype and treatment in maize.

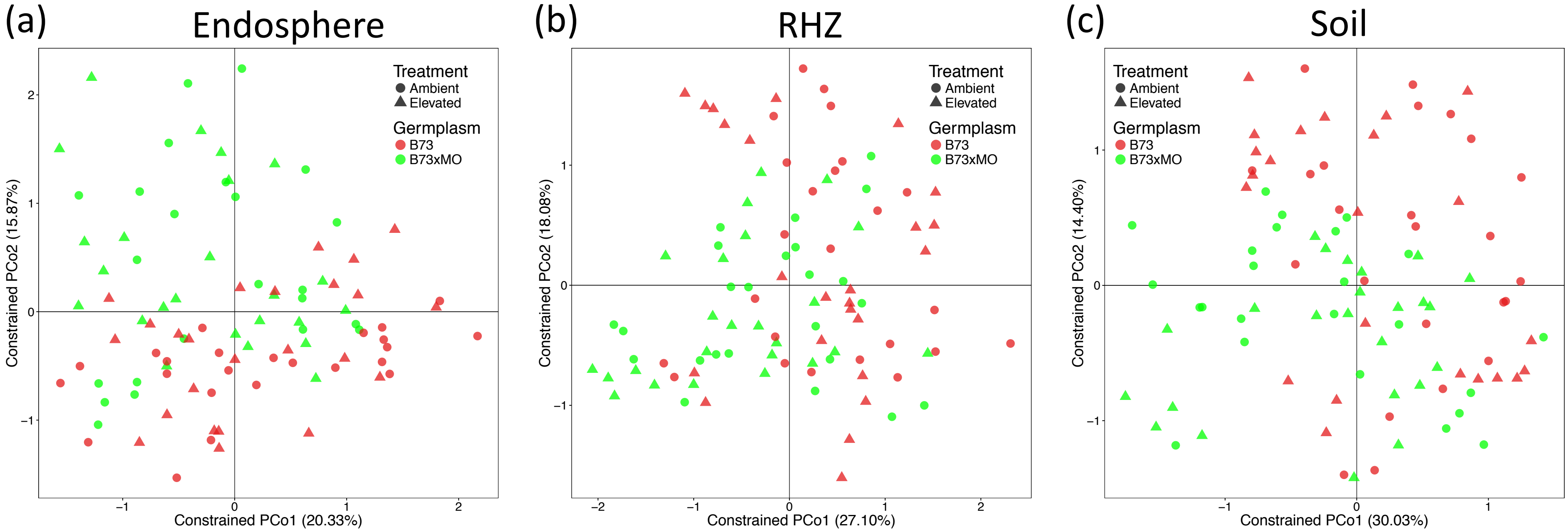

Model: capscale(formula = as.dist(gh.uuf) ~ Genotype + Treatment + Block + Condition(Ring + SoilType), data = gh.map, add = T)

Factor	Df	SumOfSqs	F	Pr(>F)
Genotype	1	0.36	1.38	0.01 **
Treatment	1	0.33	1.26	0.03 *
Block	6	1.92	1.23	0.001 ***
Residual	84	21.95		

Signif. codes: 0 ‘***’ 0.001 ‘**’ 0.01 ‘*’ 0.05 ‘.’ 0.1 ‘ ’ 1

Model: capscale(formula = as.dist(gh.uuf) ~ Genotype + Treatment + Block + Condition(Ring + SoilType), data = gh.map, add = T)

Factor	Df	SumOfSqs	F	Pr(>F)
Genotype	1	0.30	1.61	0.006 **
Treatment	1	0.16	0.83	0.89
Block	6	1.39	1.24	0.003 **
Residual	85	15.82		

Signif. codes: 0 ‘***’ 0.001 ‘**’ 0.01 ‘*’ 0.05 ‘.’ 0.1 ‘ ’ 1

Model: capscale(formula = as.dist(gh.uuf) ~ Genotype + Treatment + Block + Condition(Ring + SoilType), data = gh.map, add = T)

Factor	Df	SumOfSqs	F	Pr(>F)
Genotype	1	0.32	1.90	0.002 **
Treatment	1	0.26	1.53	0.01 **
Block	6	1.65	1.62	0.001 ***
Residual	84	14.24		

Signif. codes: 0 ‘***’ 0.001 ‘**’ 0.01 ‘*’ 0.05 ‘.’ 0.1 ‘ ’ 1

Fig. S8 Microbial community composition in soil type is not significant difference by UUF matrix analysis in three sample types of maize.

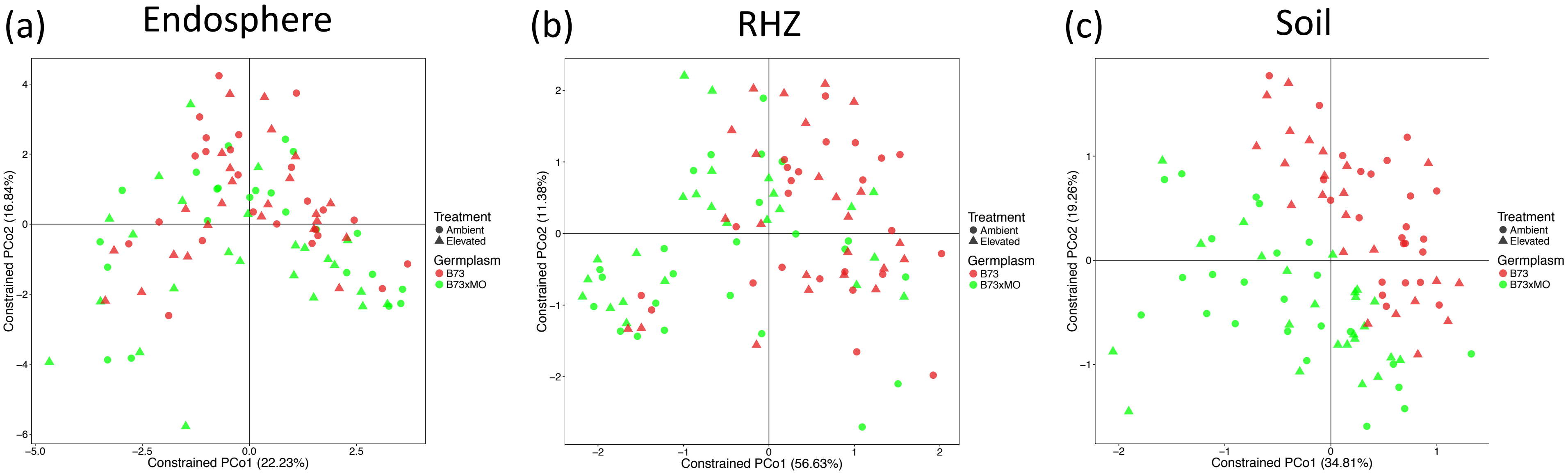

Model: capscale(formula = as.dist(gh.wuf) ~ Genotype + Treatment + Block + SoilType + Condition(Ring), data = gh.map, add = T)

Factor	Df	SumOfSqs	F	Pr(>F)
Genotype	1	1.469	1.5796	0.043 *
Treatment	1	0.924	0.9939	0.434
Block	6	7.415	1.3290	0.011 *
SoilType	1	1.237	1.3308	0.125
Residual	84	78.109		

Signif. codes: 0 ‘***’ 0.001 ‘**’ 0.01 ‘*’ 0.05 ‘.’ 0.1 ‘ ’ 1

Model: capscale(formula = as.dist(gh.wuf) ~ Genotype + Treatment + Block + SoilType + Condition(Ring), data = gh.map, add = T)

Factor	Df	SumOfSqs	F	Pr(>F)
Genotype	1	1.1442	5.0882	0.001 ***
Treatment	1	0.1566	0.6963	0.778
Block	6	1.9160	1.4201	0.021 *
SoilType	1	0.2830	1.2585	0.210
Residual	85	19.1145		

Signif. codes: 0 ‘***’ 0.001 ‘**’ 0.01 ‘*’ 0.05 ‘.’ 0.1 ‘ ’ 1

Model: capscale(formula = as.dist(gh.wuf) ~ Genotype + Treatment + Block + SoilType + Condition(Ring), data = gh.map, add = T)

Factor	Df	SumOfSqs	F	Pr(>F)
Genotype	1	0.5582	5.0889	0.001 ***
Treatment	1	0.1830	1.6685	0.027 *
Block	6	1.4529	2.2077	0.001 ***
SoilType	1	0.1250	1.1392	0.292
Residual	84	9.2134		

Signif. codes: 0 ‘***’ 0.001 ‘**’ 0.01 ‘*’ 0.05 ‘.’ 0.1 ‘ ’ 1

Fig. S9 Microbial communities composition is not significantly different between soil types by WUF analysis in endosphere, RHZ and soil.

Fig. S9 continued

(d) Endosphere

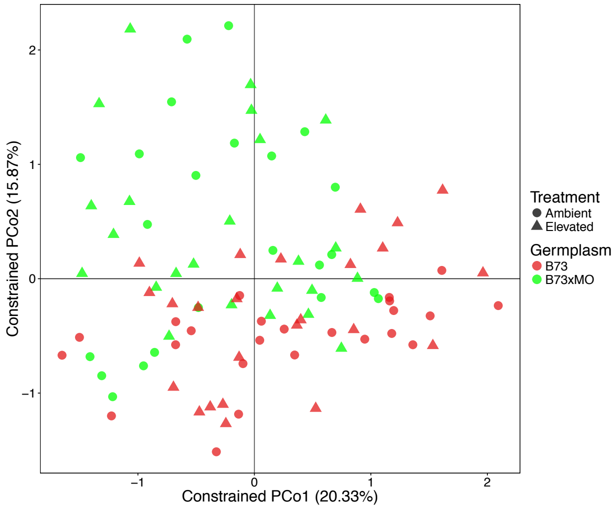

Model: capscale(formula = as.dist(gh.uuf) ~ Genotype + Treatment + Block + SoilType + Condition(Ring), data = gh.map, add = T)

Factor	Df	SumOfSqs	F	Pr(>F)
Genotype	1	0.3576	1.3685	0.004 **
Treatment	1	0.3332	1.2749	0.021 *
Block	6	1.9794	1.2625	0.001 ***
SoilType	1	0.2757	1.0551	0.289
Residual	84	21.9505		

Signif. codes: 0 '***' 0.001 '**' 0.01 '*' 0.05 '.' 0.1 ' ' 1

(e) RHZ

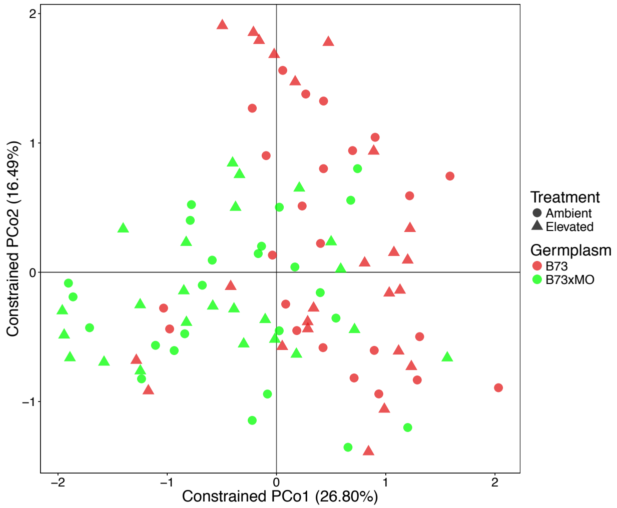

Model: capscale(formula = as.dist(gh.uuf) ~ Genotype + Treatment + Block + SoilType + Condition(Ring), data = gh.map, add = T)

Factor	Df	SumOfSqs	F	Pr(>F)
Genotype	1	0.3617	1.9440	0.001 ***
Treatment	1	0.1557	0.8366	0.893
Block	6	1.3842	1.2398	0.005 **
SoilType	1	0.1815	0.9755	0.437
Residual	85	15.8169		

Signif. codes: 0 '***' 0.001 '**' 0.01 '*' 0.05 '.' 0.1 ' ' 1

(f) Soil

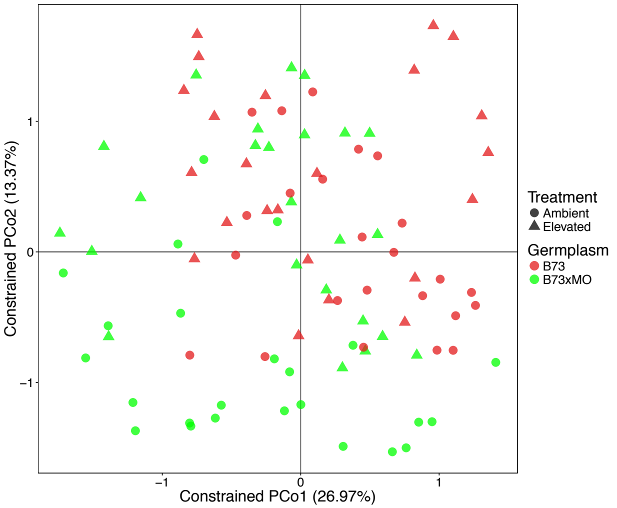

Model: capscale(formula = as.dist(gh.uuf) ~ Genotype + Treatment + Block + SoilType + Condition(Ring), data = gh.map, add = T)

Factor	Df	SumOfSqs	F	Pr(>F)
Genotype	1	0.3403	2.0075	0.001 ***
Treatment	1	0.2689	1.5863	0.005 **
Block	6	1.6804	1.6523	0.001 ***
SoilType	1	0.1940	1.1445	0.152
Residual	84	14.2385		

Signif. codes: 0 '***' 0.001 '**' 0.01 '*' 0.05 '.' 0.1 ' ' 1

Fig. S9 Microbial communities composition is not significantly different between soil types by UUF analysis in endosphere, RHZ and soil.

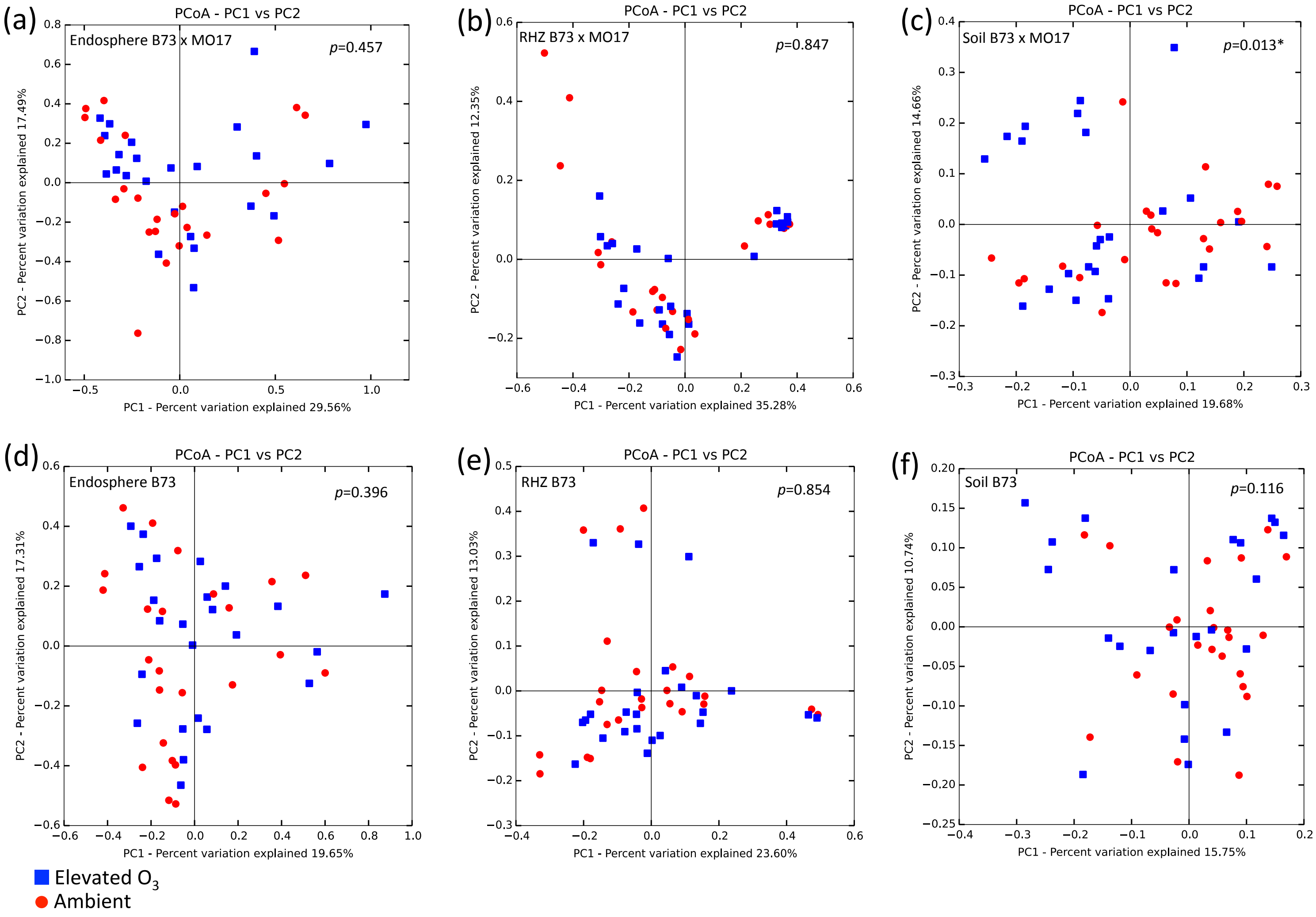

Fig. S10 Root associated microbial communities are significantly influenced by elevated ozone in soil of B73 x MO17 and root of B73 of maize. Principal coordinate analysis (PCoA) using the WUF matrix show that the microbial community composition was significantly different in ambient O_3 and elevated O_3 in soil of B73 x MO17 (p = 0.013).

Fig. S10 continued

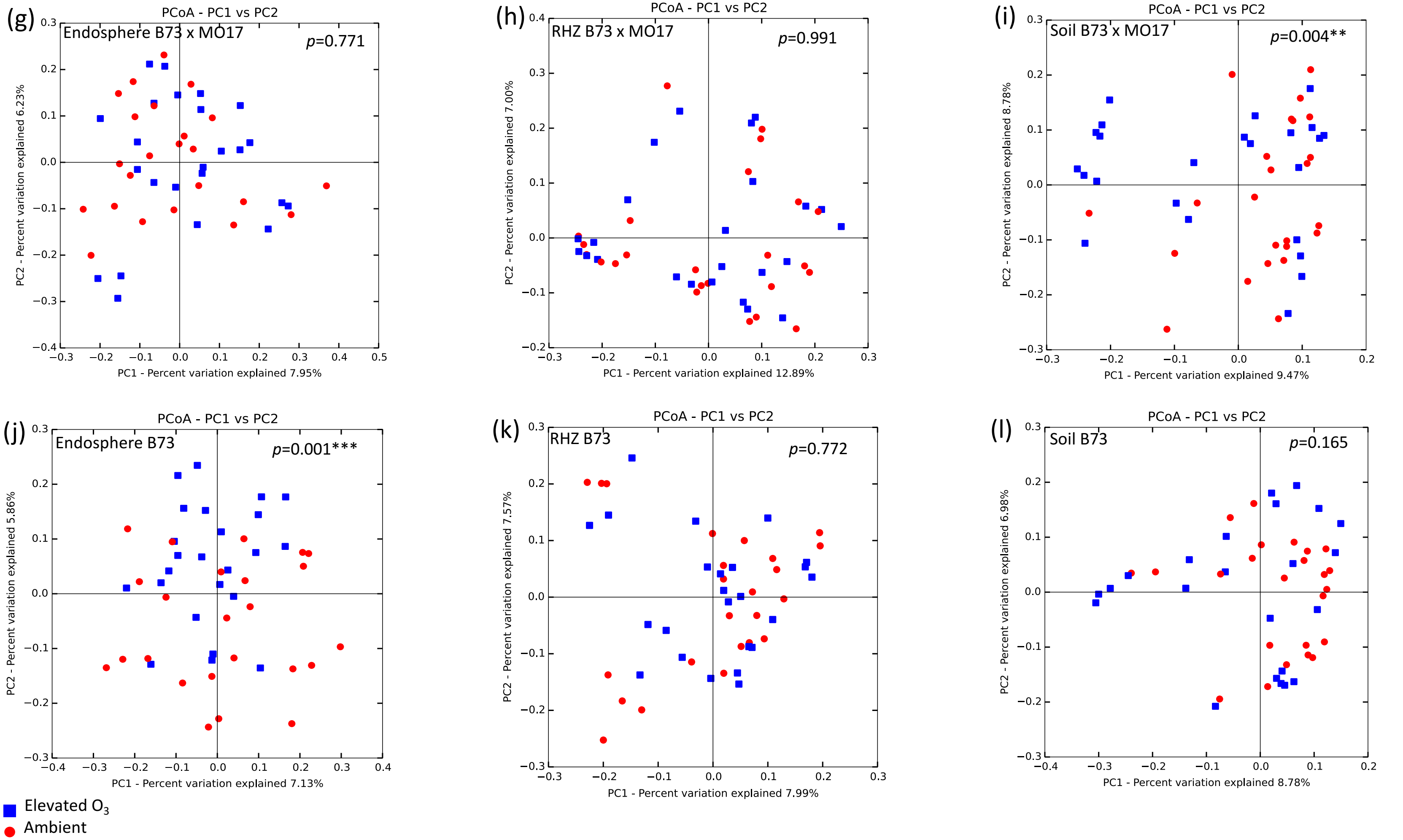

Fig. S10 Root associated microbial communities are significantly influenced by elevated ozone in soil of B73 x MO17 and root of B73 of maize. Principal coordinate analysis (PCoA) using the UUF matrix show there was significantly different microbial community composition between ambient O_3 and elevated O_3 in soil of B73 x MO17 (c, p=0.004) and endosphere of B73 (d, p=0.001), but not in: (a) endosphere of B73 x MO17 (p=0.771); (b) RHZ of B73 x MO17 (p=0.991); (e) RHZ of B73 (p=0.772); (f) and soil of B73 (p=0.165).

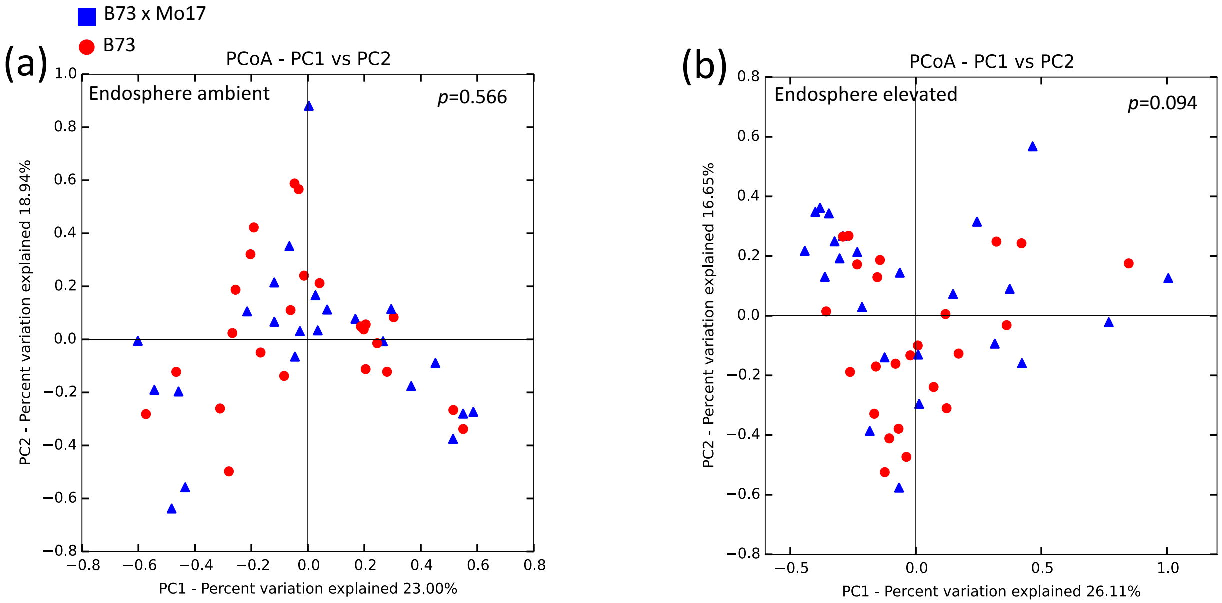

Fig. S11 Principal coordinate visualization and analysis (WUF) of genotypic effect on microbial community composition for the maize hybrid B73 X Mo17 and inbred B73 in endosphere in either ambient (a) or elevated O_3 (b). There was no significant difference of microbial communities between genotypes can be detected (p=0.566, O_3; p=0.094, eO3).

Fig. S11 continued

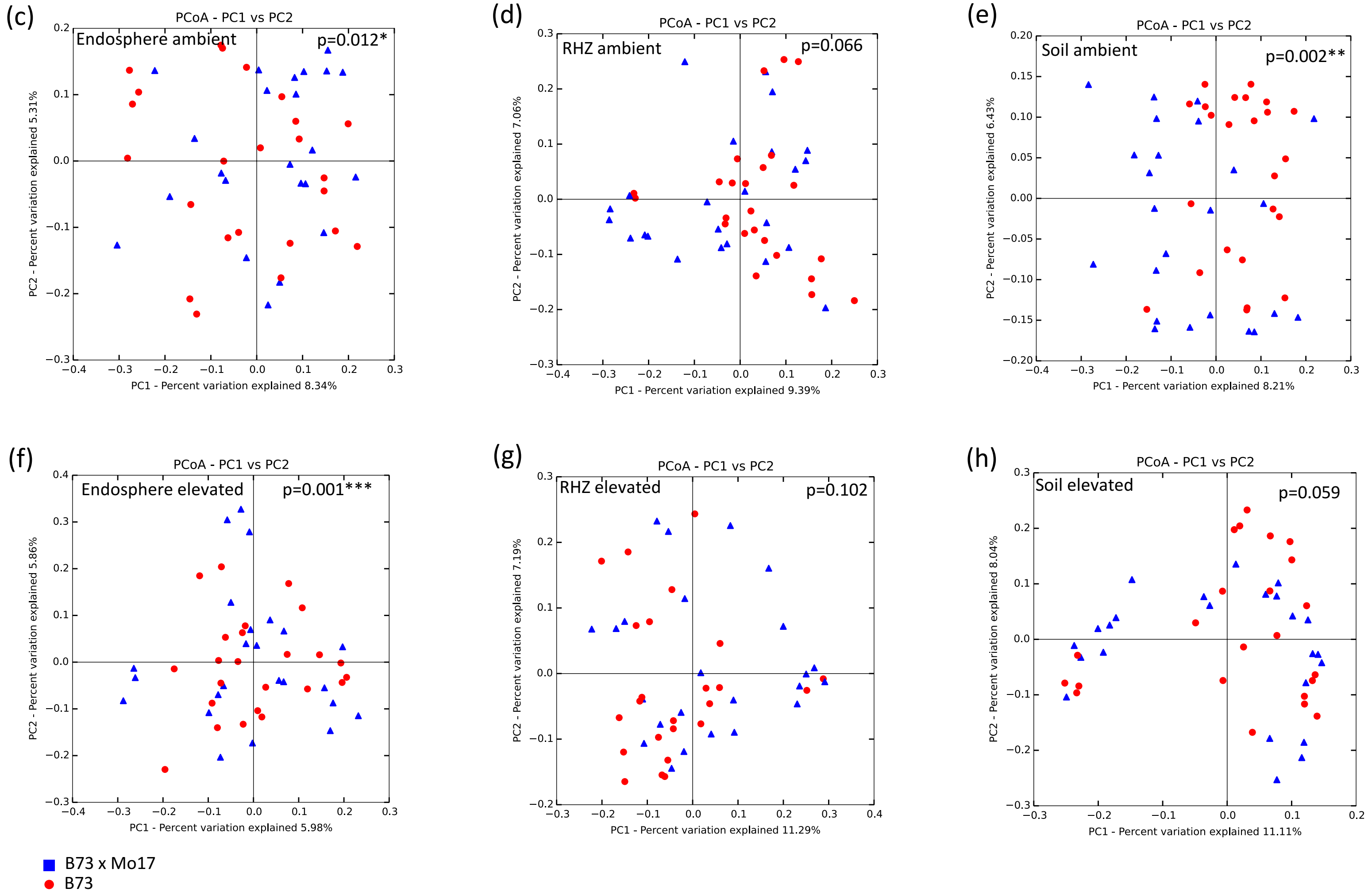

Fig. S11 Root associated microbial communities are significantly influenced by genotype of maize in endosphere using the UUF matrix. Principal coordinate analysis (PCoA) using the weighted UUF matrix show there was significantly different microbial communities between B73 x MO17 and B73 in: (c) endosphere under ambient (*p*=0.012); (e) soil under ambient (*p*=0.002); (f) endosphere under elevated (*p*=0.001); but not in: (d) RHZ under ambient (*p*=0.066); (g) RHZ (*p*=0.102) and (h) soil (*p*=0.094) under elevated. Each dot represent one sample, and the blue rectangle and red circle indicate the B73 x MO17 and B73 respectively.

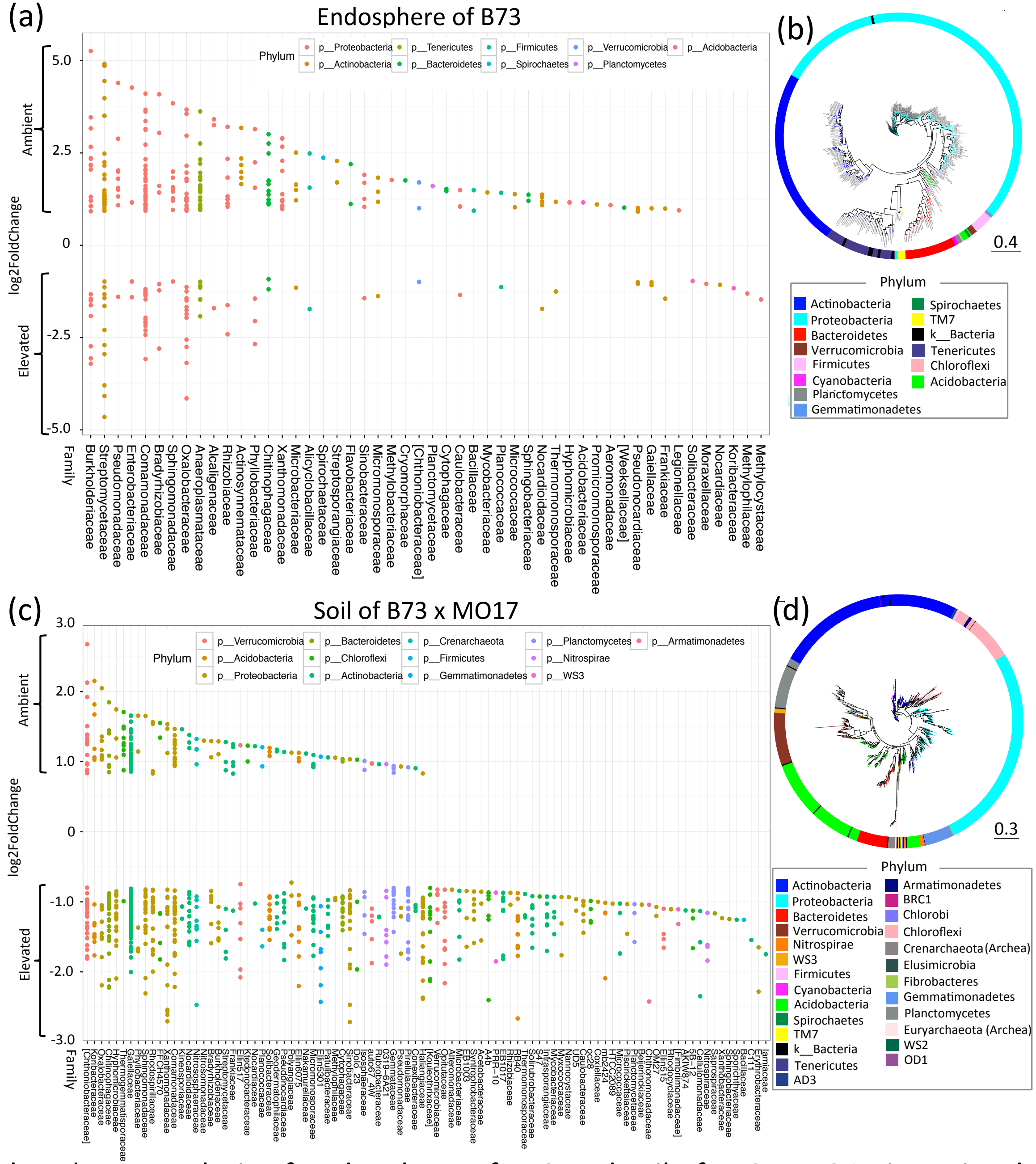

Fig. S12 Differential abundance analysis of endosphere of B73 and soil of B73 x MO17 in maize due to treatment effects. (a) OTUs that were significantly differentially abundant in aO_3 and eO_3 in endosphere of B73 and in soil of B73 x MO17 (c). (b, d) The percentage of the differentially abundant phylum differentially expressed in both ambient and elevated O_3.

Champaign County, Illinois (IL019)

Map Unit Symbol	Map Unit Name
152A	Drummer silty clay loam, 0 to 2 percent slopes
154A	Flanagan silt loam, 0 to 2 percent slopes

Treatment

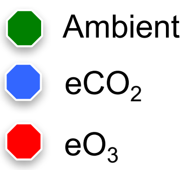

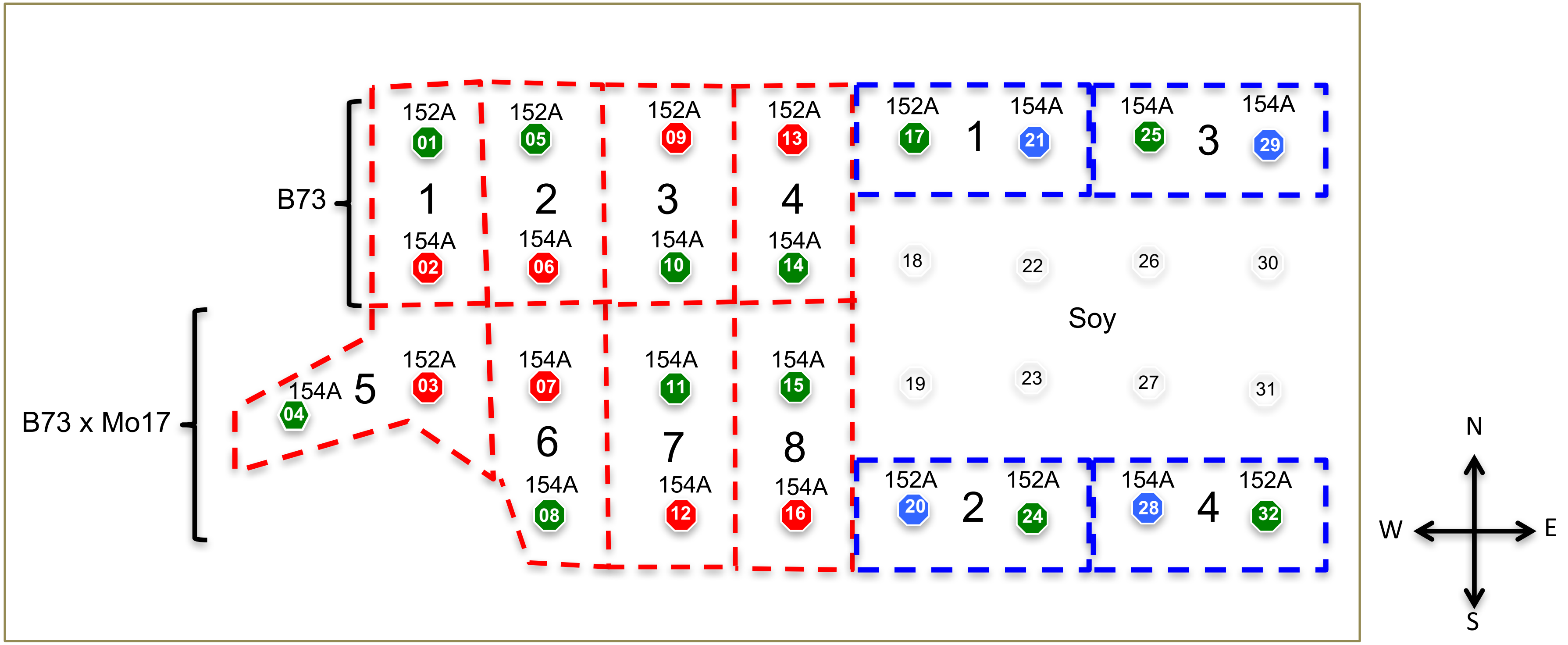

Fig. S13 Field design. The hexagonal rings indicate the treatments or control. The red dash lines shows the field where inbred and hybrid of maize were planted. The blue dash lines shows the field where soy was planted. There were eight blocks for maize and four blocks for soy. The block number is shown within the block between the rings. Two main soil types in the field are shown in the table in the top left. The soil type information for each ring is indicated above the ring.
